# MicroRNA-18a targeting of the STK4/MST1 tumour suppressor is necessary for transformation in HPV positive cervical cancer

**DOI:** 10.1371/journal.ppat.1008624

**Published:** 2020-06-18

**Authors:** Ethan L. Morgan, Molly R. Patterson, Emma L. Ryder, Siu Yi Lee, Christopher W. Wasson, Katherine L. Harper, Yigen Li, Stephen Griffin, G. Eric Blair, Adrian Whitehouse, Andrew Macdonald

**Affiliations:** 1 School of Molecular and Cellular Biology, Faculty of Biological Sciences, University of Leeds, Leeds, West Yorkshire, U.K, United Kingdom; 2 Astbury Centre for Structural Molecular Biology, University of Leeds, Leeds, West Yorkshire, U.K, United Kingdom; 3 Leeds Institute of Medical Research, Faculty of Medicine and Health, University of Leeds, Leeds, West Yorkshire, U.K, United Kingdom; University of Wisconsin-Madison, UNITED STATES

## Abstract

Human papillomaviruses (HPV) are a major cause of malignancy worldwide. They are the aetiological agents of almost all cervical cancers as well as a sub-set of other anogenital and head and neck cancers. Hijacking of host cellular pathways is essential for virus pathogenesis; however, a major challenge remains to identify key host targets and to define their contribution to HPV-driven malignancy. The Hippo pathway regulates epithelial homeostasis by down-regulating the function of the transcription factor YAP. Increased YAP expression has been observed in cervical cancer but the mechanisms driving this increase remain unclear. We found significant down-regulation of the master Hippo regulatory kinase STK4 (also termed MST1) in cervical disease samples and cervical cancer cell lines compared with healthy controls. Re-introduction of STK4 inhibited the proliferation of HPV positive cervical cells and this corresponded with decreased YAP nuclear localization and decreased YAP-dependent gene expression. The HPV E6 and E7 oncoproteins maintained low STK4 expression in cervical cancer cells by upregulating the oncomiR miR-18a, which directly targeted the *STK4* mRNA 3’UTR. Interestingly, miR-18a knockdown increased STK4 expression and activated the Hippo pathway, significantly reducing cervical cancer cell proliferation. Our results identify STK4 as a key cervical cancer tumour suppressor, which is targeted via miR-18a in HPV positive tumours. Our study indicates that activation of the Hippo pathway may offer a therapeutically beneficial option for cervical cancer treatment.

## Author summary

HPVs are the causative agents of ~5% of human cancers. Better understanding of the mechanisms by which these viruses deregulate cellular signalling pathways may offer therapeutic options for HPV-associated malignancies. The transcription factor YAP is active in cervical cancer but the mechanisms controlling its activation remain unclear. YAP is negatively regulated and sequestered in the cytoplasm through activation of the Hippo pathway. We discovered that expression of the master Hippo kinase, STK4 (also termed MST1), is reduced in HPV positive cervical cell lines and cervical disease samples. Low STK4 levels were maintained by the HPV oncogenes through up-regulation of miR-18a, which targeted the *STK4* mRNA 3’UTR. Re-introduction of STK4 or bypassing miR-18a-dependent regulation de-activated YAP-driven transcription and reduced cell proliferation. Thus, our study identifies a novel interplay between HPV oncogenes and the STK4 tumour suppressor and identifies the Hippo pathway as a target for therapeutic intervention in HPV-associated malignancies.

## Introduction

Human papillomaviruses (HPV) are a major cause of ano-genital cancers, accounting for 99.9% of all cervical cancer cases, and are linked to an increasing number of head and neck carcinomas [1–3]. The majority of HPV-associated cancers are associated with infection by two high-risk (HR) types; HPV16 and HPV18, but other HPV types can also cause cancer. The main drivers of transformation in HPV-mediated cancers are the E6 and E7 oncoproteins. These oncoproteins interact with multiple host cell factors to manipulate cellular processes and promote transformation [4–7].

The Hippo signalling pathway controls organ and tissue homeostasis across diverse species, through regulation of cellular proliferation and apoptosis [8–10]. In mammals, the core Hippo pathway is a kinase cascade comprised of the sterile 20-like family kinases serine/threonine protein kinase 4 (STK4) (also known as MST1) and STK3 (also known as MST2), the large tumour suppressors (LATS1 and LATS2) and the adaptor proteins Mps One Binder kinase activator (MOBs) and Salvador homologue 1 (SAV1) [11]. Activation of these kinases results in serine phosphorylation of the major Hippo targets yes-associated protein (YAP) and its paralogue the transcriptional co-activator with PDZ-binding (TAZ). This promotes their interaction with 14-3-3 proteins resulting in cytoplasmic sequestration or phosphorylation-dependent proteasomal degradation via the ubiquitin proteasome system. However, in their non-phosphorylated state, YAP and TAZ traffic to the nucleus where they partner with TEAD transcription factors to regulate the expression of genes necessary for cell proliferation, differentiation and survival [12].

Overexpression and aberrant activation of YAP, likely caused by deregulation of the Hippo pathway, is observed in most cancers including breast, liver and oesophageal cancer [13–15], and has been linked to shorter patient survival [16,17]. Moreover, STK4 and STK3 have been established as tumour suppressor proteins. In mice, ablation of STK4/STK3 expression leads to hepatocellular carcinoma and intrahepatic cholangiocarcinoma [18]. In colorectal cancers, STK4 expression has been found to be negatively regulated by the micro-RNA miR-590-3p [19]. One recent study recommended STK4 as an early biomarker for colorectal cancer and as a marker of poor prognosis[20].

Stabilisation of YAP has recently been observed in cervical cancer cells [21]. This is mediated by the HPV E6 oncoprotein and results in increased expression of the epidermal growth factor receptor (EGFR) ligand, amphiregulin (AREG), which in turn was found to further inhibit the Hippo pathway [21]. Furthermore, the E6 protein from the β genus HPV type 8 can induce YAP-dependent gene transcription, and this may be through attenuating the activity of the Hippo kinase LATS [22].

Clearly, manipulation of Hippo signalling appears vital for HPV pathogenesis; however, the molecular mechanisms remain unclear. Moreover, the Hippo pathway consists of many proteins beyond YAP, and the importance of these in the context of cervical cancer biology remains unknown.

To understand how the Hippo pathway may be de-regulated in cervical cancer, we analysed the expression and activation status of critical components of the pathway in a panel of cervical cancer cell lines. STK4 protein levels were selectively decreased in HPV positive (HPV+) cervical cancer cells when compared with normal human keratinocytes (NHKs) or HPV negative (HPV-) cervical cancer cells. Importantly, STK4 levels were also lower in cytology samples from patients with cervical disease and in cervical cancer samples compared to healthy controls. Reintroduction of STK4 or STK3, into HPV+ cancer cells inhibited their proliferation, migration and invasion. Mechanistically, we discovered that in HPV+ cervical cancer cells HPV E6 and E7 expression led to suppression of *STK4* mRNA levels and this was achieved through an increase in the mature form of miR-18a, which directly targeted the 3’UTR of the *STK4* mRNA. In summary, we have identified that HPV+ cervical cancer cells upregulate an oncogenic miRNA (oncomiR) to incapacitate the tumour suppressive Hippo pathway and increase YAP-dependent gene expression and proliferation.

## Results

### STK4 expression is decreased in HPV+ cervical cancer cell lines

**Fig 1:**
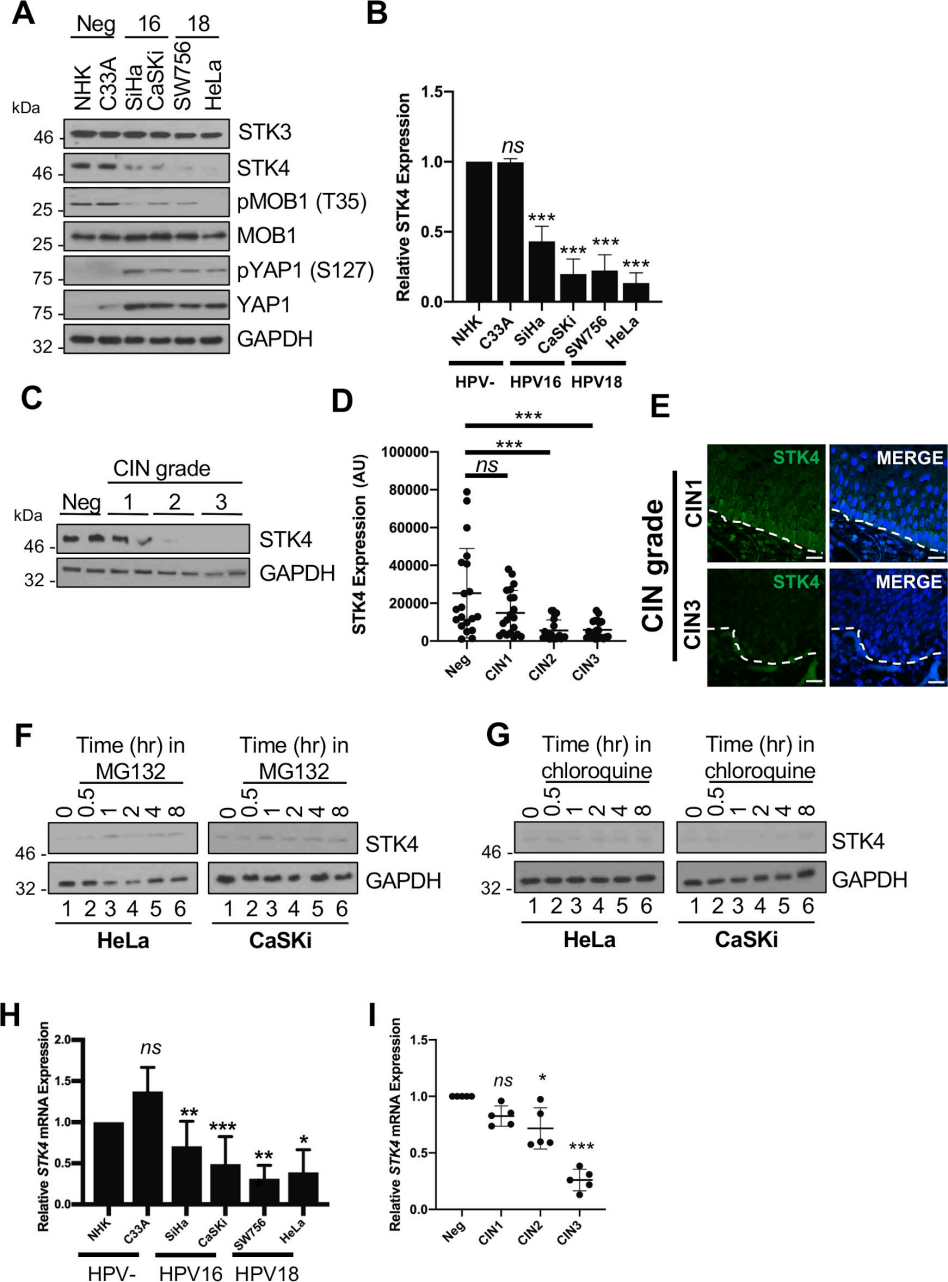
STK4 expression is down regulated in HPV+ cervical cancer. **A)** Representative western blot analysis of a panel of HPV- and HPV+ cervical cancer cell lines for components of the Hippo pathway. GAPDH served as a loading control. **B)** Densitometry analysis of STK4 expression in **A** from four independent experiments. SiHa p = 0.0087 and CaSKi p = 0.0044; SW756 p = 0.0052 and HeLa p = 0.0018; C33A p = 0.44 when compared to NHK. **C)** Representative western blot from cytology samples of CIN lesions of increasing grade analysed for STK4 expression. GAPDH served as a loading control. **D)** Scatter plot of densitometry analysis of a panel of cytology samples. Twenty samples from each clinical grade (Neg, CIN 1–3) were analysed by western blot and densitometry analysis was performed for STK4 expression. CIN1 not significant; CIN2 p = 0.001; CIN3 p = 0.001 when compared with neg samples. **E)** Representative immunofluorescence analysis of tissue sections from cervical lesions representing LSIL and HSIL. Sections were stained for STK4 expression (green) and nuclei were visualised using DAPI (blue). Images were acquired using identical exposure times. Scale bar, 20 μm. **F-G)** Representative western blot of proteasome inhibitor MG132 (**F**) and lysosome inhibitor chloroquine (**G**) dose response in HeLa and CaSKi cells. Cell lysates were probed for the expression of STK4. GAPDH was used as a loading control (N = 3). **H)** qPCR analysis of *STK4* transcript levels in a panel of HPV- and HPV+ cervical cancer cell lines. *U6* expression was used as a loading control (n = 3). SiHa p = 0.01 and CaSKi p = 0.004; SW756 p = 0.001 and HeLa p = 0.05; C33A p = 0.12 when compared to NHK. **I)** qPCR analysis of *STK4* transcript levels from different CIN grades (n = 5 from each grade). *U6* expression was used as a control. CIN1 not significant; CIN2 p = 4.6x10^-7^; CIN3 p = 1.1x10^-5^ when compared to neg samples. Error bars represent the mean +/- standard deviation. *P<0.05, **P<0.01, ***P<0.001 (Student’s t-test).

We investigated how the Hippo pathway is regulated in cervical cancer cells by first measuring the protein expression and enzymatic activity of essential components of the pathway. Compared with normal primary human keratinocytes (NHKs) or the HPV negative (HPV-) cervical cancer cell line C33A, levels of total YAP and the phosphorylated form of YAP (pYAP1 S127) were significantly increased in HPV positive (HPV+) cells (Fig 1A, quantified in 1B), consistent with previous studies [21]. In contrast, levels of the STK4 tumour suppressor protein were significantly reduced in HPV16+ and HPV18+ cell lines compared to NHK cells, whereas in HPV- C33A cells STK4 protein expression was similar to that observed in NHK cells (Fig 1A). As expected, the reduction in STK4 protein in the HPV+ cell lines was accompanied by a decrease in phosphorylation of the STK4 substrate MOB1 (Fig 1A).

Importantly, this pattern of STK4 expression was mirrored in cervical liquid based cytology samples from a cohort of HPV16+ patients representing cervical intraepithelial neoplasia (CIN) progression, which commonly precedes cervical cancer. CIN1 is thought to represent a transient HPV infection, while CIN3 represents clinically significant HPV infection that may progress to cervical cancer [23]. Cytology samples from patients identified as HPV- and showing no signs of cervical disease served as controls in these experiments. By western blot, STK4 protein levels decreased during disease progression through the CIN2 and CIN3 grades (Fig 1C and quantified in 1D). These findings were corroborated using STK4 immunofluorescence in sections of cervical tissue from low-grade CIN1 and high-grade CIN3 samples, revealing a marked decrease in STK4 expression in CIN3 in agreement with western blot data (Fig 1E). Together, these data demonstrate that STK4 protein expression is down-regulated in HPV+ cervical disease.

### *STK4* mRNA levels are reduced in HPV+ cervical cancer cells

To understand how STK4 protein levels were reduced in HPV+ cervical cancer cells, we first measured whether inhibition of protein degradation restored its expression, as HPV E6 has previously been shown to stabilise YAP, a downstream target of STK4 [21]. HeLa and CaSKi cells were incubated with inhibitors of proteasomal (MG132) or lysosomal (Chloroquine) degradation for 8 hours and STK4 protein levels measured by western blot. Neither inhibitor had a significant impact on STK4 expression over the course of the experiment (Fig 1F and 1G). Next, we probed for changes in *STK4* mRNA levels. Using qRT-PCR, we observed that *STK4* mRNA levels were selectively decreased in both HPV16+ and HPV18+ cells compared with the NHK and C33A cells (Fig 1H). Crucially, a reduction in *STK4* mRNA levels was also observed in cytology samples from patients with CIN2 and CIN3 cervical disease (Fig 1I). To provide additional evidence of *STK4* down-regulation in patients, we mined available microarray databases [24]. From these we observed little change in *STK4* levels between normal cervical epithelia and low-grade squamous intraepithelial lesions (LSIL; comparable to CIN1). In contrast, we saw a significant decrease in *STK4* mRNA expression in high-grade squamous intraepithelial lesions (HSIL; comparable to CIN3) when compared to low-grade and normal cervical epithelia (S1A and S1B Fig). Building on this, we were able to show that decreased *STK4* mRNA expression is a common occurance in cervical cancers (S1C–S1E Fig).

### STK4 overexpression re-activates Hippo signalling in HPV+ cancer cells

**Fig 2:**
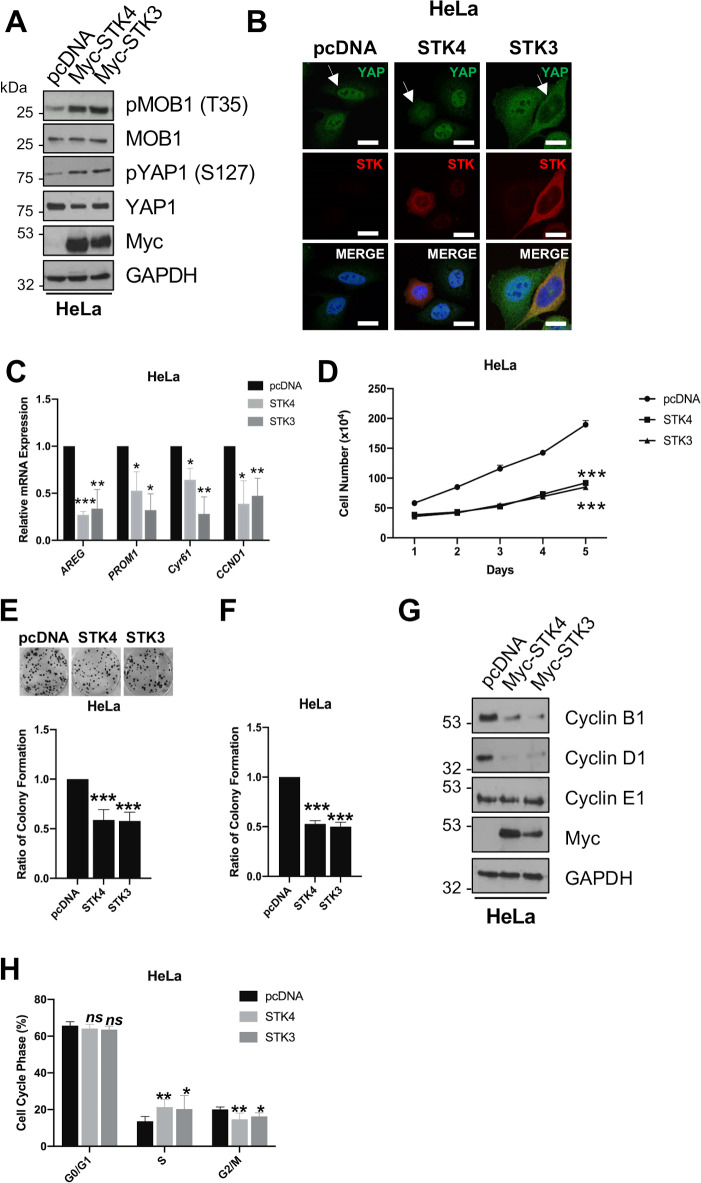
STK4/3 inhibits proliferation and cell cycle progression in HPV+ cervical cancer cells. **A)** Representative western blots of STK4/3 overexpression in HeLa cells. Lysates were analysed for the phosphorylation of the STK4/3 substrate MOB1 and the downstream target YAP. The Myc epitope was used to detect successful expression of fusion proteins. GAPDH was used as a loading control. **B)** Immunofluorescence analysis of STK4/3 overexpression in HeLa cells. Cover slips were stained for STK4/3 (red) and YAP (green). Nuclei were visualised using DAPI (blue). Images were acquired using identical exposure times. Scale bar, 20 μm. **C)** qPCR analysis of YAP-dependent genes (*AREG*, *PROM1*, *Cyr61* and *CCND1)* in HeLa cells overexpressing STK4/3. *U6* expression was used as a loading control (n = 3). **D)** Growth curve analysis of HeLa cells overexpressing STK4 or STK3 (n = 4). **E)** Colony formation assay (anchorage dependent growth) of HeLa cells overexpressing STK4 or STK3 (n = 4). **F)** Soft agar assay (anchorage independent growth) of HeLa cells overexpressing STK4 or STK3 (n = 4). **G)** Representative western blots of HeLa cells overexpressing STK4 or STK3 analysed for the expression of cyclin proteins. The Myc epitope was used to detect expression of fusion proteins. GAPDH was used as a loading control. **H)** Flow cytometric analysis of cell cycle profile of HeLa cells overexpressing STK4 or STK3. Error bars represent the mean +/- standard deviation of a minimum of three biological repeats. *P<0.05, **P<0.01, ***P<0.001 (Student’s t-test).

To assess the implications of reduced STK4 expression in HPV+ cervical cancer, STK4 and its paralogue STK3/MST2 were over-expressed in HeLa and CaSKi cells. STK3 shares 75% similarity to STK4 and the two proteins are thought to share some functions [25]. Transfection of Myc-tagged STK4 or STK3 increased MOB1 and YAP1 phosphorylation, indicating that their overexpression activated the Hippo pathway (Fig 2A and S2A Fig). By immunofluorescence microscopy, cells with re-activated Hippo pathway showed a redistribution of YAP from the nucleus to the cytoplasm (indicated by the white arrows) (Fig 2B and S2B Fig). Redistribution of YAP significantly reduced the mRNA levels of a selection of YAP-dependent genes tested including amphiregulin (*AREG)*, CD133 *(PROM1)*, cyr61 (*cyr61)* and cyclin D1 *(CCND1)* (Fig 2C and S2C Fig). Thus, overexpression of STK4/STK3 in HPV+ cervical cancer cells reconstitutes a functionally active Hippo signalling pathway capable of excluding YAP from the nucleus and reducing YAP-regulated gene expression.

### STK4 inhibits proliferation and cell cycle progression of HPV+ cancer cells

The Hippo pathway negatively regulates tumour progression in a number of cancers. Thus, it was pertinent to investigate the consequences of re-activating Hippo signalling in cervical cancer cells by the re-introduction of STK4 or STK3. Compared to controls, overexpression of either STK4 or STK3 into HeLa and CaSKi cells resulted in a significant decrease in cell proliferation (Fig 2D and S2D Fig). Additionally, expression of either protein suppressed the ability of HeLa and CaSKi cells to form anchorage-dependent and anchorage-independent colonies (Fig 2E and 2F and S2E and S2F Fig).

We assessed the levels of cyclin proteins, which drive cell proliferation, and upon re-activation of the Hippo pathway cyclin B1 and cyclin D1 protein levels were reduced whilst cyclin E1 expression remained unchanged (Fig 2G and S2G Fig). Flow cytometry analysis confirmed that cells over expressing STK4/STK3 accumulated cells in S-phase, with a corresponding decrease in the number of cells in the G2/M phases, indicating that cells over-expressing STK4/STK3 have a prolonged S phase (Fig 2H and S2H Fig). Together, these data show that Hippo re-activation results in a defect in cell proliferation and the ability of HeLa and CaSKi cells to form colonies in an anchorage dependent or independent manner, highlighting a potential tumour suppressor role for STK4/STK3 in HPV+ cervical cancer cells.

In contrast, whilst a clear increase in Hippo pathway activity was seen in HPV- C33A cells over-expressing STK4 and STK3 (S3A–S3C Fig), this had a negligible impact on cell proliferation, colony formation or cell cycle progression (S3D–S3F Fig). This suggests that the tumour suppressor activity of STK4/STK3 may be specific to HPV+ cervical cancer cells and warrants further investigation.

### Kinase activity is essential for STK4 tumour suppressor function

**Fig 3:**
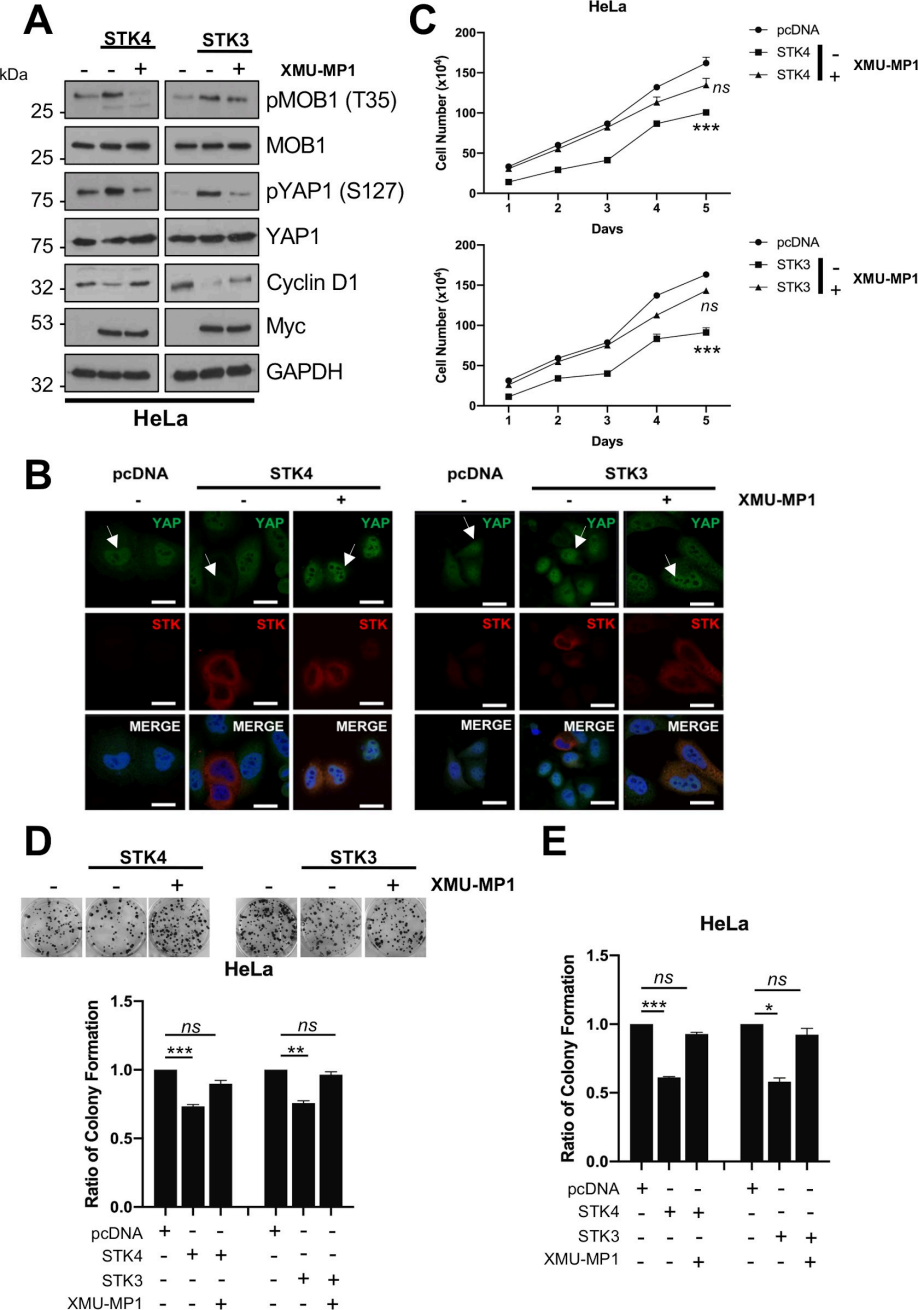
Inhibition of STK4/3 kinase activity prevents the block on proliferation and tumourigenesis. **A)** Representative western blots of STK4/3 overexpression in HeLa cells with or without treatment with XMU-MP1 for 8 hours prior to lysis. Lysates were analysed for the phosphorylation of the STK4/3 substrate MOB1, the downstream target YAP and the YAP target gene cyclin D1. The Myc epitope was used to detect fusion protein expression. GAPDH was used as a loading control. **B)** Immunofluorescence analysis of STK4/3 overexpression in HeLa cells with or without treatment with XMU-MP1 for 8 hours prior to analysis. Cover slips were stained for STK4/3 (red) and YAP1 (green). Nuclei were visualised using DAPI (blue). Images were acquired using identical exposure times. Scale bar, 20 μm. **C)** Growth curve analysis of HeLa cells overexpressing STK4/3 with or without treatment with XMU-MP1 for 8 hours prior to re-seeding (n = 3). **D)** Colony formation assay (anchorage dependent growth) of HeLa cells overexpressing STK4/3 with or without treatment with XMU-MP1 for 8 hours prior to re-seeding (n = 3). **E)** Soft agar assay (anchorage independent growth) of HeLa cells overexpressing STK4/3 with or without treatment with XMU-MP1 for 8 hours prior to re-seeding (n = 3). Error bars represent the mean +/- standard deviation of a minimum of three biological repeats. *P<0.05, **P<0.01, ***P<0.001 (Student’s t-test).

**Fig 4:**
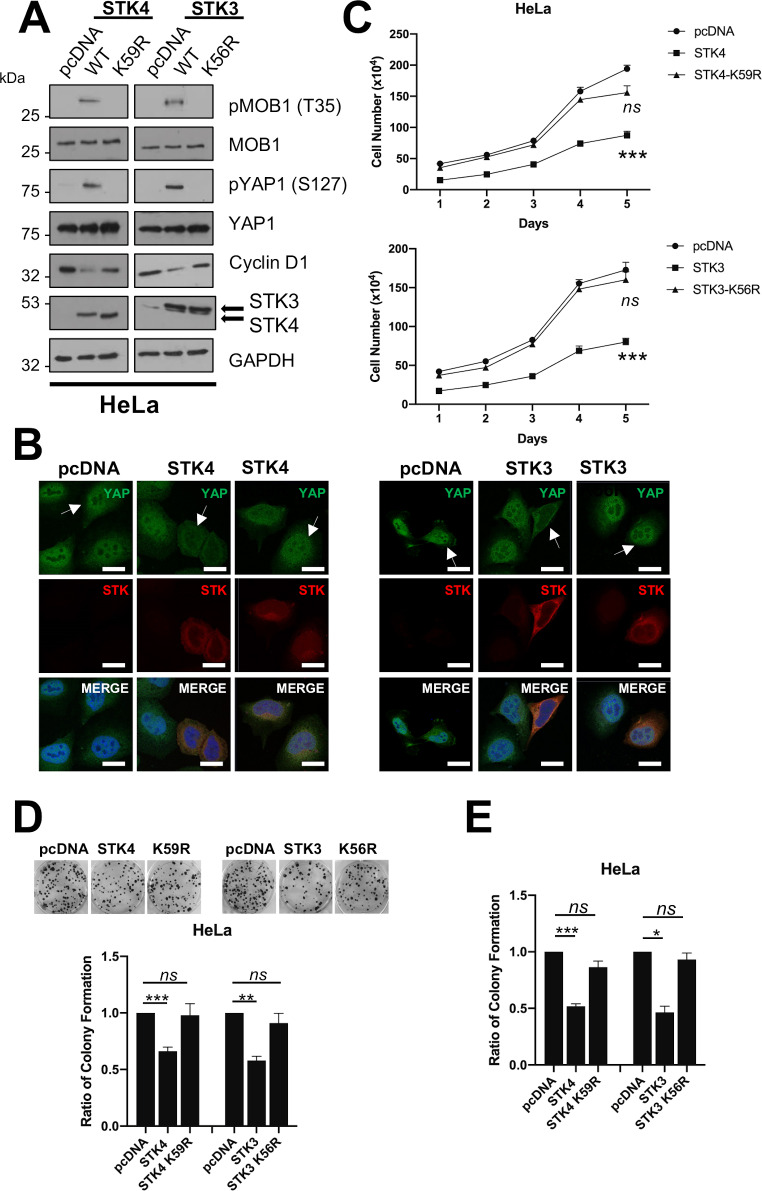
Kinase null STK4/3 mutants do not inhibit proliferation and tumourigenesis. **A)** Representative western blots of STK4/3 or kinase dead (KD) STK4/3 overexpression in HeLa cells. Lysates were analysed for the phosphorylation of the STK4/3 substrate MOB1, the downstream target YAP and the YAP target gene cyclin D1. Antibodies against STK4/3 were used to detect successful expression. GAPDH was used as a loading control. **B)** Immunofluorescence analysis of STK4/3 or KD STK4/3 overexpression in HeLa cells. Cover slips were stained for STK4/3 (red) and YAP1 (green). Nuclei were visualised using DAPI (blue). Images were acquired using identical exposure times. Scale bar, 20 μm. **C)** Growth curve analysis of HeLa cells overexpressing STK4/3 or KD STK4/3 (n = 3). **D)** Colony formation assay (anchorage dependent growth) of HeLa cells overexpressing STK4/3 or KD STK4/3 (n = 3). **E)** Soft agar assay (anchorage independent growth) of HeLa cells overexpressing STK4/3 or KD STK4/3 (n = 3). Error bars represent the mean +/- standard deviation of a minimum of three biological repeats. *P<0.05, **P<0.01, ***P<0.001 (Student’s t-test).

To investigate whether kinase activity was required for the anti-proliferative effects of STK4/STK3 overexpression, we tested the STK specific inhibitor XMU-MP1 [26]. Cells overexpressing STK4 or STK3 were treated with XMU-MP1 (1 μM) for 8 hours prior to analysis. At this dose and time point, no overall effects on cell viability were observed. XMU-MP1 treatment reduced MOB1 and YAP phosphorylation, indicating successful inhibition of kinase activity (Fig 3A and S4A Fig). Crucially, XMU-MP1 treatment of HeLa or CaSKi cells increased YAP nuclear localisation (Fig 3B and S4B Fig, white arrows) and increased expression of the YAP-dependent gene product cyclin D1 (Fig 3A and S4A Fig). Confirmatory experiments were performed with catalytically inactive STK4 (K59R) or STK3 (K56R). These mutants were not able to phosphorylate MOB1 (Fig 4A and S5A Fig) or redistribute YAP from the nucleus to the cytoplasm (Fig 4B and S5B Fig, white arrows) and failed to reduce cyclin D1 expression (Fig 4A and S5A Fig).

To further assess the importance of STK kinase activity on cervical cancer cell proliferation, cells overexpressing STK4 or STK3 were incubated with XMU-MP1. XMU-MP1 treatment abolished the effects on cell proliferation (Fig 3C and S4C Fig) and colony formation (Fig 3D and 3E and S4D and S4E Fig). Importantly, similar results were observed when the kinase null mutants were overexpressed in HPV+ cells (Fig 4C–4E and S4C–S4E Fig). These data demonstrate that the tumour suppressive functions observed in HPV+ cancer cells require catalytically active STK4/STK3.

### HPV oncoproteins are essential but not sufficient to inhibit *STK4* expression

**Fig 5:**
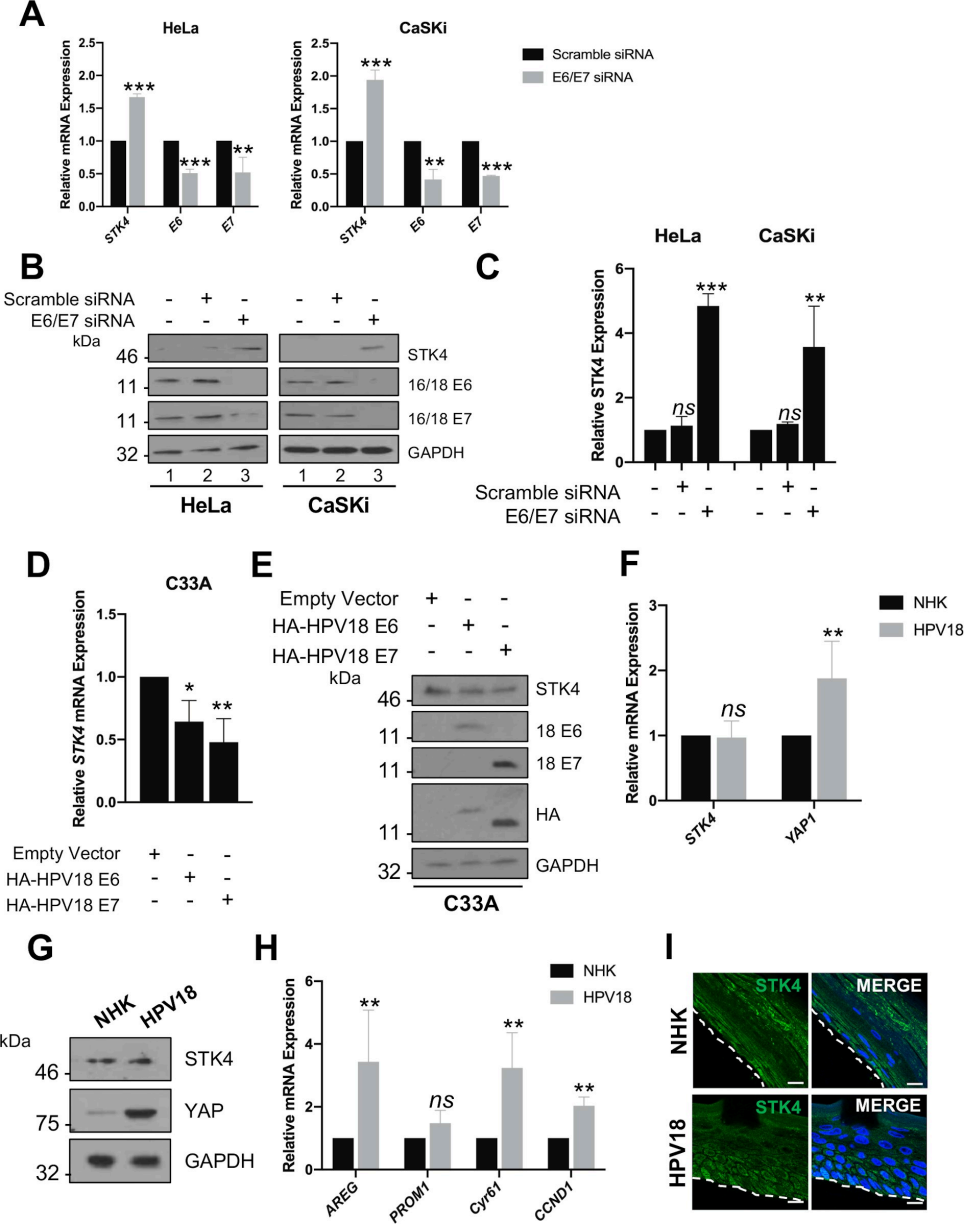
HR-HPV E6 and E7 oncoproteins suppress *STK4* expression. **A)** qPCR analysis of *STK4* transcript levels in HeLa and CaSKi cells transfected with HPV16 or HPV18 specific E6 and/or E7 siRNA, respectively. *U6* was used as a loading control (n = 3). **B)** Representative western blots of HeLa and CaSKi cells transfected with HPV16 or HPV18 specific E6 and/or E7 siRNA, respectively, and analysed for STK4 protein expression. GAPDH was used as a loading control. **C)** Quantification of **B** from three independent experiments. **D)** qPCR analysis of *STK4* transcript levels in C33A cells stably expressing HPV18 E6 or E7. *U6* was used as a loading control (n = 3). **E)** Representative western blot of C33A cells stably expressing HPV18 E6 or E7 and analysed for the expression of STK4. HPV18 E6 and E7 expression was confirmed using antibodies against E6 and E7 and the HA-tag. GAPDH was used as a loading control. **F)** qPCR analysis of *STK4* and *YAP1* expression in NHK and HPV18-containing NHK cells. *U6* was used as a loading control (n = 3). **G)** Representative western blot of STK4 and YAP from NHK and HPV18-containing NHK cells. GAPDH was used as a loading control. **H)** qPCR analysis of YAP-dependent gene expression in NHK and HPV18-containing NHK cells. *U6* was used as a loading control (n = 3). Error bars represent the mean +/- standard deviation. *P<0.05, **P<0.01, ***P<0.001 (Student’s t-test). **I)** Representative immunofluorescence analysis of sections of organotypic raft cultures of NHK and HPV18-containing NHK cells. Sections were stained for STK4 expression (green) and nuclei were visualised using DAPI (blue). Images were acquired using identical exposure times. Scale bar, 20 μm.

The E6 and E7 oncoproteins are key drivers of transformation in HPV+ cervical cancers [27]. We hypothesized that these oncoproteins were responsible for the reduction in *STK4* mRNA levels and subsequent protein expression observed in HPV+ cervical cancer. To investigate this, we employed an siRNA knockdown strategy in which HPV18+ HeLa or HPV16+ CaSKi cells were transfected with a pool of siRNAs targeting E6 and E7 [28]. Transfection of these siRNAs led to a reduction of both the mRNA and protein expression of HPV E6 and E7 in each cell line (Fig 5A and 5B). Oncoprotein depletion significantly increased *STK4* mRNA expression (Fig 5A) and this corresponded with increased STK4 protein levels in both HPV16+ and HPV18+ cervical cancer cell lines (Fig 5B and quantification in Fig 5C). These data demonstrate that the HPV oncogenes are required for the down-regulation of *STK4* gene expression observed in cervical cancer cells.

Inhibition of E6/E7 expression has previously been shown to induce apoptosis and cellular senescence [29]. Additionally, STK4 is activated in response to apoptotic signals and activation of the DNA damage response (DDR) [30]. To assess if activation of these pathways was responsible for the induction of *STK4* expression, we treated HeLa cells with staurosporine (apoptosis; [31]), H_2_0_2_ (DNA damage; [32]) or low dose etoposide (DNA damage induced senescence; [33]) and assessed *STK4* mRNA expression. As STK4 can be cleaved upon genotoxic stress [34], we did not assess STK4 protein expression. None of the treatments resulted in a significant change in *STK4* mRNA expression, suggesting that the effect of E6/E7 depletion on STK4 expression was not due to secondary impacts on the induction of apoptosis, the DDR or DNA damage induced senescence (S6A–S6C Fig). Furthermore, over-expression of p53, which occurs upon knockdown of E6 [28], also failed to increase *STK4* mRNA expression (S6D Fig).

To determine if ectopic expression of HPV oncoproteins was able to reduce *STK4* expression, we generated HPV- C33A cells stably expressing HPV18 E6 or E7. Overexpression of either E6 or E7 resulted in a reduction in *STK4* mRNA expression by approximately 40% and 50%, respectively (Fig 5D); however, subsequent STK4 protein expression was only reduced by around 15% with each oncoprotein (Fig 5E). This indicates that viral oncoprotein expression by itself may not be sufficient to reduce STK4 expression. To assess this further, we analysed STK4 levels in primary human keratinocytes stably harbouring the HPV18 genome, which we have previously shown express both the E6 and E7 oncoproteins [5,28]. Whilst YAP levels (Fig 5F and 5G) and YAP-dependent transcriptional activity (Fig 5H) was significantly higher in keratinocytes containing HPV18 compared to control cells, we were surprised to observe no significant changes in STK4 expression (Fig 5F and 5G). This finding was not an artefact of growing the keratinocytes in monolayer culture, as stratification of cultures by organotypic raft culture which induces keratinocyte differentiation and recapitulates the productive HPV life cycle also caused little change in STK4 levels (Fig 5I). Taken together, these data suggest that HPV E6/E7 expression is essential but not sufficient to down-regulate STK4 expression. They also confirm that YAP is active during the productive HPV life cycle, not just in HPV disease, but suggest that YAP activation is mediated by a mechanism independent of the loss of STK4.

### miR-18a is upregulated in HPV+ cervical cancer and directly targets *STK4* mRNA

**Fig 6:**
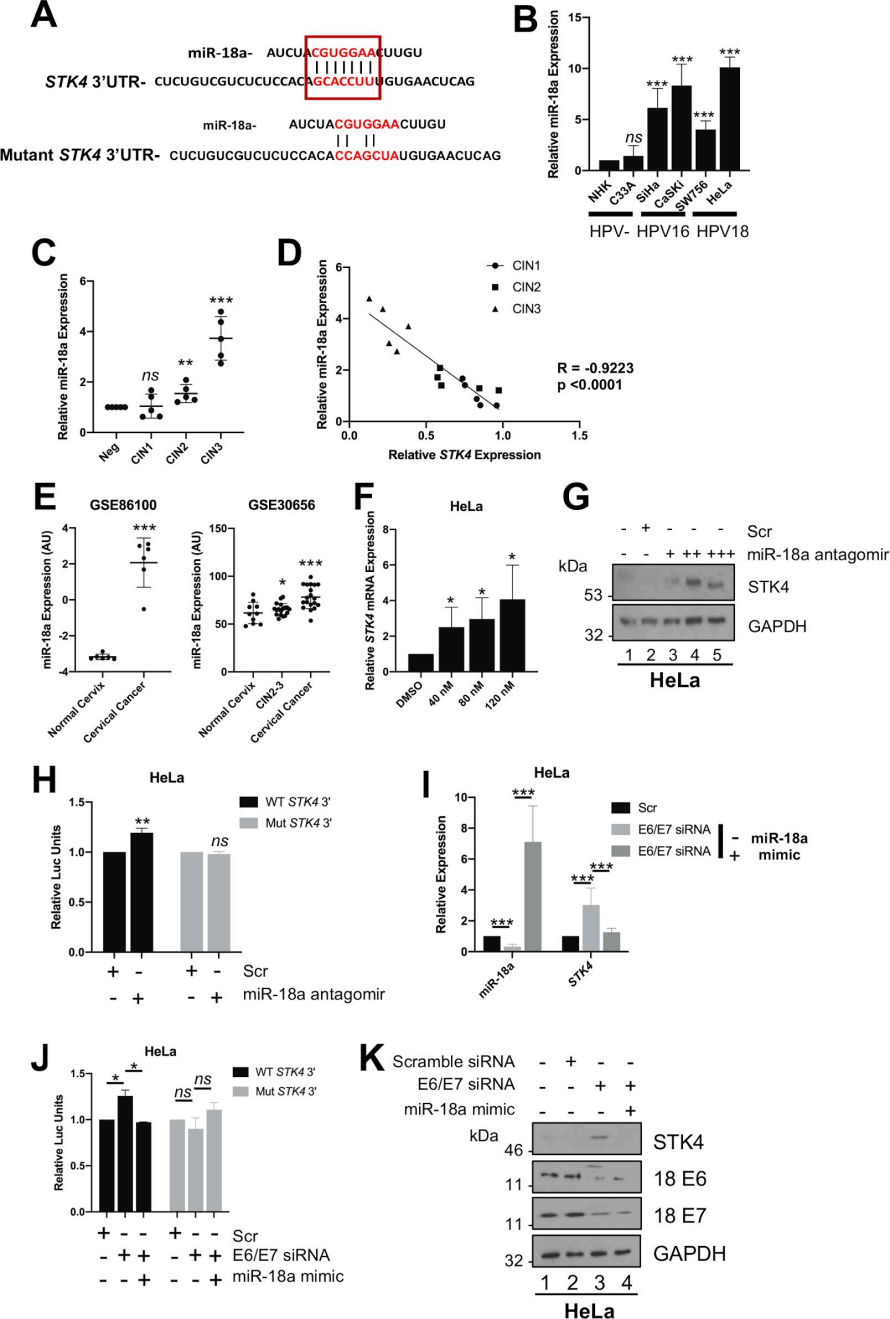
miR-18a is upregulated in cervical cancer and directly targets *STK4*. **A)** Schematic of *STK4* 3’UTR showing miR-18a binding site and mutant miR-18a binding site. **B)** miScript analysis of miR-18a levels in a panel of cervical cancer cell lines. snORD68 expression was used as a loading control (n = 3). **C)** qPCR analysis of *STK4* transcript levels from different CIN grades (n = 5 from each grade). snORD68 expression was used as a loading control. **D)** Graph showing correlation between miR-18a and STK4 expression in matched patient samples of cervical disease (n = 15). **E)** Scatter plot of data from GSE86100 (normal cervix (n = 26) and cervical cancer (n = 30)) and GSE30656 (normal cervix (n = 10), high-grade cervical intraepithelial neoplasia (CIN2-3) (n = 18) and cervical carcinoma (n = 19)). Arbitrary expression values for the microRNA expression of miR-18a in each sample were plotted. **F)** qPCR analysis of *STK4* transcript levels in HeLa cells with increasing doses of miR-18a antagomir (n = 3). **G)** Representative western blot of HeLa cells with increasing doses of miR-18a antagomir. Cell lysates were probed for the expression of STK4. GAPDH was used as a loading control. **H)** Luciferase reporter assays from HeLa cells cotransfected with miR-18a antagomir and either a wild-type *STK4* 3’UTR reporter plasmid or a mutant that lacks the putative miR-18a binding site. Data presented are relative to an internal firefly luciferase control (n = 3). **I)** miScript analysis of miR-18a levels in HeLa transfected with specific E6/E7 siRNA with or without a miR-18a mimic. snORD68 was used as a loading control (n = 3). **J)** Luciferase reporter assays from HeLa cells cotransfected with specific E6/E7 siRNA, with or without a miR-18a mimic, and either a wild-type *STK4* 3’UTR reporter plasmid or a mutant that lacks the putative miR-18a binding site. Data presented are relative to an internal firefly luciferase control (n = 3). **K)** Representative western blot of HeLa cells cotransfected with specific E6/E7 siRNA, with or without a miR-18a mimic. Cell lysates were probed for the expression of STK4. GAPDH was used as a loading control. Error bars represent the mean +/- standard deviation. *P<0.05, **P<0.01, ***P<0.001.

Both HPV E6 and E7 can modulate the microRNA (miRNA) network as a mechanism to control host gene expression [35–37]. The oncomiR miR-18a, a member of the oncogenic miR-17-92 cluster, is highly expressed in cervical cancer and has been shown to contribute to the proliferative and invasive phenotype, through ill-defined mechanisms [38,39]. Bioinformatic analysis of the *STK4* mRNA 3’UTR region revealed a putative miR-18a binding site (Fig 6A). We first confirmed that miR-18a was up-regulated in our panel of cervical cancer cell lines by miScript analysis. Compared with NHK and C33A cells, levels of mature miR-18a were increased in both HPV16+ and HPV18+ cell lines (Fig 6B). In line with this, analysis of cervical cytology samples highlighted a significant correlation between increased miR-18a levels and cervical disease progression, which inversely correlated with *STK4* expression (Fig 6C and 6D). In agreement with our data, analysis of available microarray datasets showed a significant increase in miR-18a expression in cervical cancer compared to normal tissue (Fig 6E).

To inhibit miR-18a, cells were transfected with a miR-18a antagomir and *STK4* mRNA levels were assessed by qRT-PCR. Inhibition of miR-18a led to a dose-dependent increase in *STK4* mRNA and protein levels in HeLa cells (Fig 6F and 6G). To address if *STK4* is a direct target of miR-18a, HeLa and CaSKi cells were transfected with the miR-18a antagomir and a reporter plasmid containing a renilla luciferase sequence fused to the *STK4* 3’UTR. Cells transfected with the miR-18a antagomir showed increased luciferase levels, suggesting that miR-18a directly regulates *STK4* gene expression in HPV+ cervical cancer cells (Fig 6H and S7A Fig). To confirm that miR-18a acted through the putative miR-18a binding site identified in the *STK4* 3’ UTR, we performed site directed mutagenesis to generate a 3’UTR sequence lacking complementarity to miR-18a (Fig 6A). In contrast to the wild-type, luciferase expression from the mutant *STK4* 3’ UTR plasmid was unaffected by the miR-18a antagomir (Fig 6H and S7A Fig). These data suggest that in HPV+ cervical cancer cells, miR-18a directly regulates *STK4* gene expression through binding to the miR-18a binding site within the *STK4* 3’ UTR.

Given that *STK4* expression is regulated by HPV E6/E7, we hypothesised that miR-18a expression may also in some way be governed by the viral oncoproteins. We depleted E6 and E7 by siRNA and noted a significant decrease in miR-18a expression, corresponding to the increase in *STK4* expression previously observed (Fig 6I and S7B Fig). Knockdown of E6 and E7 also caused an increase in luciferase levels driven by the wild-type *STK4* 3’ UTR luciferase plasmid but not from the miR-18a binding site mutant plasmid (Fig 6J and S7C Fig). Importantly, re-introduction of a miR-18a mimic in the E6 and E7 knockdown cells ablated the increase in luciferase levels (Fig 6J and S7C Fig), *STK4* mRNA expression (Fig 6I and S7B Fig) and STK4 protein expression (Fig 6K and S7D Fig). Interestingly, transfection of a miR-18a antagomir into HPV- C33A cells did not affect *STK4* mRNA expression (S8A Fig), suggesting that miR-18a does not regulate the Hippo pathway in these cells. Together, these data demonstrate that the E6 and E7 mediated increase in miR-18a regulates *STK4* gene expression by directly targeting the *STK4* 3’ UTR.

### Inhibition of miR-18a reduces cervical cancer cell proliferation by activating the STK4—Hippo pathway

**Fig 7:**
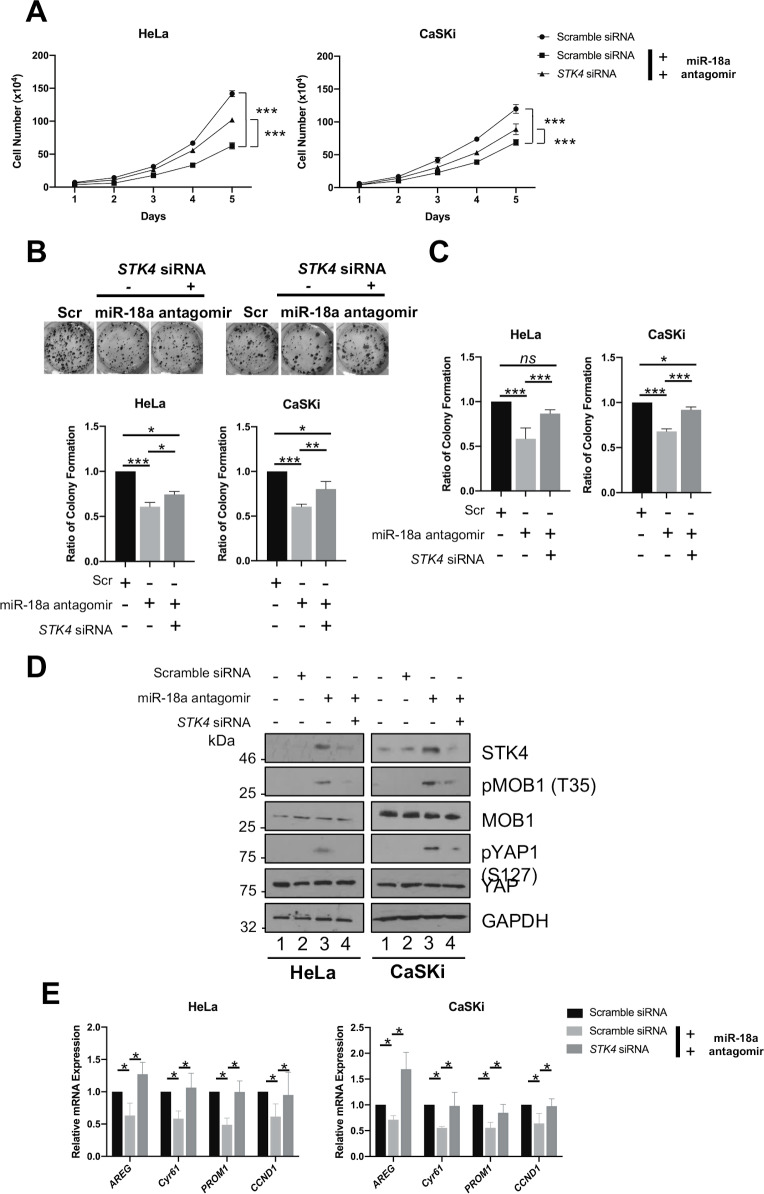
The proliferative defect upon miR-18a inhibition is partially dependent on increased STK4 protein expression. **A)** Growth curve analysis of HeLa and CaSKi cells transfected with miR-18a inhibitor, with or without *STK4* siRNA (n = 3). **B)** Colony formation assay (anchorage dependent growth) of HeLa and CaSKi cells transfected with miR-18a inhibitor, with or without *STK4* siRNA (n = 3). **C)** Soft agar assay (anchorage independent growth) of HeLa and CaSKi cells transfected with miR-18a inhibitor, with or without *STK4* siRNA (n = 3). **D)** Representative western blots of HeLa and CaSKi cells transfected with miR-18a inhibitor, with or without *STK4* siRNA. Lysates were analysed for STK4, the phosphorylation of the STK4 substrate MOB1, the downstream target YAP and the YAP target gene cyclin D1. GAPDH was used as a loading control. **E)** qPCR analysis of YAP dependent gene transcripts in HeLa and CaSKi cells transfected with miR-18a inhibitor, with or without *STK4* siRNA (n = 3). Error bars represent the mean +/- standard deviation of a minimum of three biological repeats. *P<0.05, **P<0.01, ***P<0.001.

We analysed whether miR-18a has a role in regulating cell proliferation, given that STK4 re-introduction inhibits HPV+ cell proliferation. Introduction of the miR-18a antagomir led to a significant decrease in cell proliferation and the ability of these cells to form colonies in both an anchorage dependent and independent manner (Fig 7A–7C). Since miR-18a is documented to affect expression of a number of tumour suppressive genes, we investigated if the effects of miR-18a inhibition were dependent on its targeting of *STK4* expression. For this, we employed an siRNA knockdown strategy in which HeLa and CaSKi cells were transfected with a pool of siRNA targeting *STK4* alongside addition of the miR-18a antagomir. Critically, knockdown of *STK4* partially restored cervical cancer cell proliferation, suggesting that the observed effects of miR-18a inhibition are in part dependant on the inhibition of *STK4* gene expression (Fig 7A–7C).

Next, we analysed the effects of miR-18a on activation of the Hippo pathway. Transfection of the miR-18a antagomir led to an increase in both MOB1 and YAP phosphorylation (Fig 7D), and these increases were reduced in cells treated with the *STK4* specific siRNA. Additionally, knockdown of *STK4* expression in combination with inhibition of miR-18a activity restored expression of YAP-dependent genes compared to inhibition of miR-18a alone (Fig 7E). Together, these data demonstrate that miR-18a activity promotes proliferation in HPV+ cervical cancer by suppressing *STK4* gene expression to inactivate the Hippo pathway.

## Discussion

Despite the current availability of prophylactic vaccines against HPV, there are no specific anti-viral therapies for HPV-associated cancers. Therefore, research is still required to understand the virus-host interactions found in these cancers and to identify the host factors essential for transformation in order to identify targets for therapeutic intervention. Indeed, targeting host cell proteins by small molecule inhibitors has been utilised as a potential antiviral therapy for a number of diverse viruses, including those associated with cancer development [40–42]. The Hippo pathway is deregulated in many cancers [43]. As the canonical activator of the Hippo pathway STK4 serves as a negative regulator of tumourigenesis, consequently its expression is down-regulated in several cancers [18,20]. In this study, we used a combination of patient samples and cancer cell lines to provide the first evidence that the loss of the STK4 is associated with the YAP-mediated proliferation of cervical cancer cells. Levels of STK4 were selectively reduced in HPV+ cervical cancer lines and these findings were supported by clinical data, showing that *STK4* expression negatively correlates with cervical disease progression and is markedly down regulated in cervical cancer. Crucially, re-introduction of STK4 expression in HPV+ cervical lines caused a significant decrease in cell proliferation. In contrast, overexpression of STK4 had minimal impact on the growth of HPV- cervical cancer cells. Given that we observed increased Hippo pathway signalling in all cell lines overexpressing STK4, our results suggest that HPV+ cancer cells are particularly sensitive to re-activation of this pathway.

The Hippo pathway converges to negatively regulate the transcription factor YAP, a key driver of cell proliferation that is activated in most solid tumours, including cervical cancer [21]. Both YAP protein levels and activity were markedly increased in HPV+ cervical cancer cells compared to HPV- cervical cancer cell lines and primary keratinocytes. Importantly, STK4 overexpression in HPV+ cervical cancer cells led to an inhibition of YAP function, as seen by the increased YAP phosphorylation and a corresponding decrease in cell proliferation. These findings indicate that in HPV+ cervical cancer cells YAP activity is sensitive to the levels of STK4 and that STK4 mediated signalling, most likely through the canonical Hippo pathway, is a predominant negative regulator of YAP in these cells. In stark contrast, whilst YAP expression and transcriptional activity were also significantly up-regulated in keratinocytes harbouring HPV18 genomes compared to uninfected donor controls, levels of STK4 were unaffected by the presence of HPV18. These cell-based studies are supported by patient data, which show little change in STK4 expression in LSIL biopsies or CIN1 cytology samples compared to healthy cervical tissue. In patient samples, a significant down-regulation of STK4 was only observed in CIN2 and CIN3, as well as in cervical cancer. These findings appear paradoxical, since previous studies show that YAP is predominantly nuclear in HPV16 containing raft cultures, indicative of high YAP activity in these cells [44]. We interpret these findings to suggest that active YAP may be essential for both productive HPV infection and to drive proliferation in HPV-associated cancers. However, the mechanisms employed by HPV to circumvent the normal regulatory barriers on enhanced YAP activity may differ between productive infection and disease. In primary keratinocytes activation of YAP appears to be independent of a reduction in STK4 expression, whilst re-expression of this protein in cervical cancer cells is deleterious to cell proliferation. The necessity for loss of STK4 expression most likely develops during disease progression and may reflect differences in the signalling circuitry between primary keratinocytes and transformed cells. Further studies will be essential in order to fully dissect the role of YAP-dependent signalling during the HPV life cycle and in the progression to cancer development.

The decrease in STK4 levels observed was dependent on HPV oncoprotein expression. These proteins have well defined roles to inhibit the function of critical tumour suppressors. Whilst inactivation of pRb and p53 by E6 and E7, respectively, is the defining characteristic of these proteins [45,46], it is now clear that their loss alone is not sufficient to initiate cervical cancer [47,48]. These findings highlight the complex nature of virus-mediated transformation and suggest that perturbation of multiple host factors is required for cervical cancer progression. We showed that loss of either oncoprotein from cervical cancer cells resulted in the re-appearance of *STK4*; however, ectopic expression of the oncoproteins in HPV- cells was not able to fully recapitulate the loss of STK4 observed in cervical cancer cells. Moreover, perturbation of cellular processes manipulated by E6 and E7 such as the level of p53, DNA damage or cell stress did not alter *STK4* expression. Thus, we conclude that in cervical cancer cells the HPV oncoproteins are necessary but not sufficient for the loss of STK4. Again, these data fit with our observations in primary keratinocytes, where we observe robust expression of E6 and E7 [5,28], but little impact on STK4 levels.

To begin to understand how STK4 is controlled in HPV transformed cells we focussed on miRNAs, given that *STK4* expression can be regulated by miRNAs [49–51] and that the HPV oncoproteins have been reported to alter expression of host miRNAs to promote transformation [35]. We focused on miR-18a, as this can regulate *STK4* gene expression in prostate cancer and miR-18a is highly expressed in invasive cervical cancer cells [39,49]. We confirmed that miR-18a is overexpressed in cervical cancer cells and in clinical biopsies of cervical cancer. Interestingly, miR-18a expression was increased in CIN2 and CIN3, but not CIN1; this is the opposite trend to STK4. In matched samples, miR-18a expression inversely correlated with *STK4* mRNA expression. Our data is also in line with previous reports demonstrating that miR-18a expression is significantly increased in cervical cancer, but not in CIN [52]. Furthermore, miR-18a expression is not differentially regulated in keratinocytes harbouring either the HPV16 or HPV18 genomes [35]. Whilst miR-18a expression may not be significantly altered, it would still be useful to determine if it is required during the productive HPV life cycle.

Crucially, inhibition of miR-18a using an antagomir resulted in the restoration of *STK4* gene expression, activation of the Hippo pathway and a subsequent defect in proliferation in HPV+ cells. In contrast, miR-18a had no effect on *STK4* expression in HPV- C33A cells, which would indicate cell specific effects of the miRNA. Although mIR-18a has been shown to target a number of potential tumour suppressor proteins we found that silencing of STK4 partially restored the proliferative ability of HPV+ cells. This would indicate that in these cells STK4 is the predominant target, although it would be useful to identify the additional mIR-18a targets that regulate cervical cancer cell proliferation.

**Fig 8:**
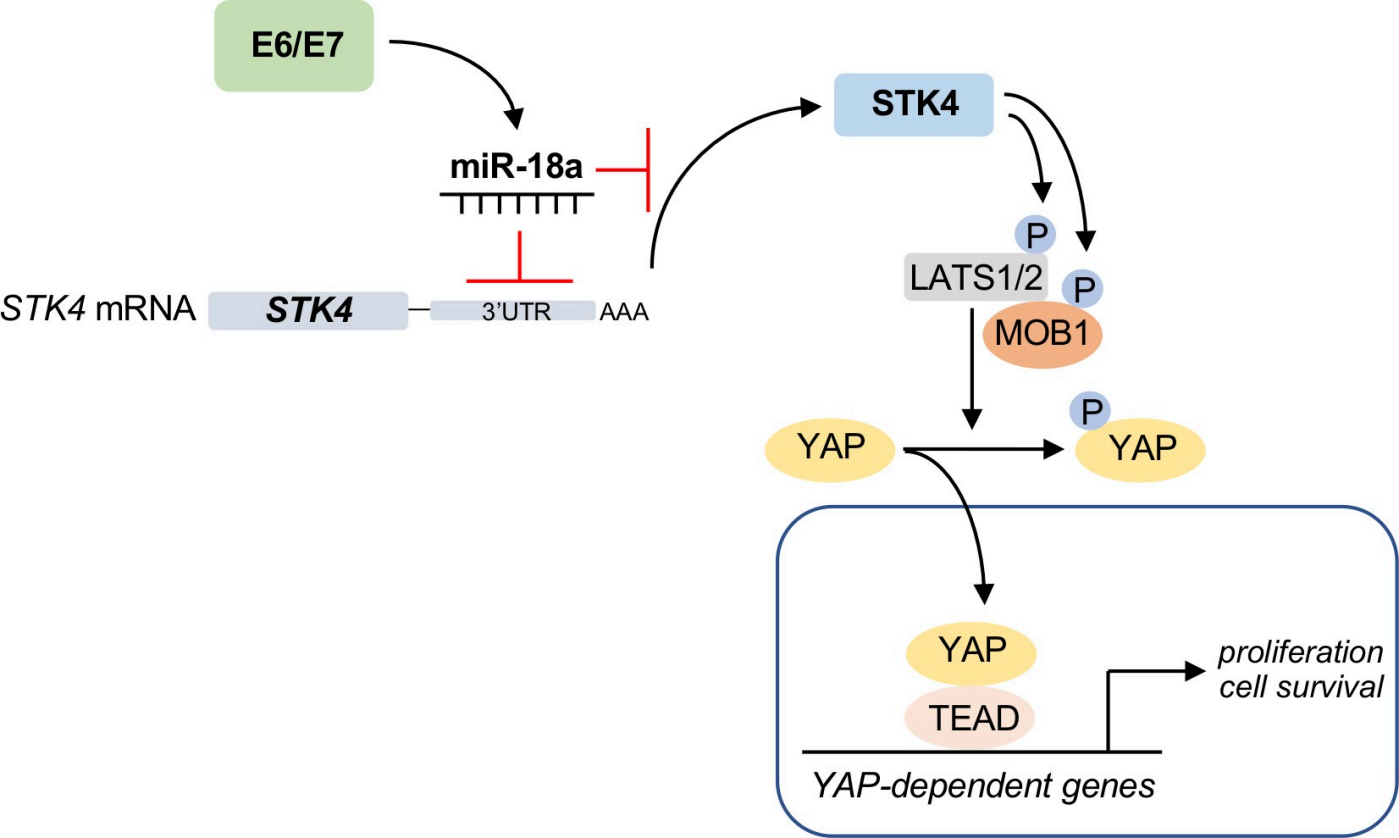
Schematic of HPV-mediated deregulation of the Hippo pathway. Expression of HPV oncoproteins in concert with changes to the host cell upregulates miR-18a expression, which directly targets the *STK4* 3’UTR to inhibit *STK4* expression. Loss of STK4 correlates with inhibition of the Hippo pathway and activation of the YAP transcription factor, culminating in increased YAP-dependent transcription. Image was generated using BioRENDER.com.

To the best of our knowledge, this study is the first to describe the presence of a signalling pathway in which HPV oncoproteins participate in a process to up-regulate miR-18a in order to suppress expression of the STK4 tumour suppressor (Fig 8). Whilst suppression of STK4 is essential for the growth of HPV+ cervical cancer cells, it appears non-essential in driving YAP-dependent signalling in primary keratinocytes harbouring the HPV18 genome. A picture is emerging in which the HPV life cycle and pathogenesis are dependent on manipulation of the Hippo pathway, which can be targeted through multiple means [53]. Therefore, targeting of this pathway may serve as a new therapeutic target in the treatment of HPV-associated malignancies.

## Materials and methods

### Cervical cytology samples

Cervical cytology samples were obtained from the Scottish HPV Archive (http://www.shine/mvm.ed.ac.uk/archive.shtml), a biobank of over 20,000 samples designed to facilitate HPV research. The East of Scotland Research Ethics Service has given generic approval to the Scottish HPV Archive as a Research Tissue Bank (REC Ref 11/AL/0174) for HPV related research on anonymised archive samples. Samples are available for the present project though application to the Archive Steering Committee (HPV Archive Application Ref 0034). RNA and protein were extracted from the samples using Trizol as described by the manufacturer (ThermoFischer Scientific, USA) and analysed as described.

### HPV positive biopsy samples

Archival paraffin-embedded cervical biopsy samples were obtained with informed consent. Subsequent analysis of these samples was performed in accordance with approved guidelines, which were approved by Glasgow Royal Infirmary: RN04PC003. HPV presence was confirmed by PCR using GP5+/GP6+ primers.

### Cell culture

HeLa (HPV18+ cervical epithelial adenocarcinoma cells), SW756 (HPV18+ cervical squamous carcinoma cells), SiHa (HPV16+ cervical squamous carcinoma cells), CaSKi (HPV16+ cervical squamous carcinoma cells) and C33A (HPV- cervical squamous carcinoma) cells obtained from the ATCC were grown in DMEM supplemented with 10% FBS (ThermoFischer Scientific, USA) and 50 U/mL penicillin. Primary normal human keratinocytes (NHKs) were maintained in serum free medium (SFM; GIBCO, UK) supplemented with 25 μg/mL bovine pituitary extract (GIBCO, UK) and 0.2 ng/mL recombinant EGF (GIBCO, UK). All cells were cultured at 37°C and 5% CO_2_. Primary keratinocytes and HPV18-containing keratinocytes were created and cultured as previously described [28]. Cells were negative for Mycoplasma during this investigation. Cell identify was confirmed by STR profiling.

### Plasmids and inhibitors

pIC-STK4-Myc and pIC-STK3-Myc were provided by Steve Cohen, LMCB, Singapore. pJ3M-STK4 K59R (Addgene plasmid # 12204) and pJ3H-STK3 K56R (Addgene plasmid # 12206) were purchased from Addgene (Cambridge, MA, USA). pcDNA-p53 was a kind gift from Olivier Terrier (CIRI, France) The psiCheck2 plasmid was provided by Dr James Boyne (University of Huddersfield, UK). The *STK4* 3’UTR was identified used NCBI data using the AceView program. It was subsequently amplified from HeLa cells and cloned into psiCheck2 using XhoI and NotI. Codon optimized HPV18 E6 and E7 sequences were cloned into pMSCV-N-HA-IRES-Puro as XhoI-NotI fragments (plasmid kindly provided by Elizabeth White, University of Pennsylvania). The small molecule inhibitor XMU-MP1 was purchased from Selleckchem and used at a final concentration of 1 μM[26]. Staurosporine, etoposide and hydrogen peroxide (H_2_O_2_) were purchased from Calbiochem and used at the concentrations indicated.

### siRNA and antagomir

The HPV16 E6 siRNA was purchased form Santa Cruz Biotechnology (USA) and had the following sequence: 5’–UGUGUACUGCAAGCAACAG– 3’. The HPV18 E6 siRNAs were purchased from Dharmacon (GE Healthcare, USA) and had the following sequences: 5’–CUAACACUGGGUUAUACAA– 3’ AND 5’–CTAACTAACACTGGGTTAT– 3’. The HPV16 and HPV18 E7 siRNA were as previously described [54,55]. The STK4 siRNAs were purchased from Qiagen (FlexiTube GeneSolution GS6789 for STK4; SI02637145, SI02637138, SI00086870, SI00086863).

### Transfections and mammalian cell lysis

Transfection of plasmid DNA was performed with a DNA to Lipofectamine^®^ 2000 (ThermoFischer) ratio of 1:2.5. 48 hr post transfection, cells were lysed in lysis buffer for western blot analysis, processed for RNA extraction or reseeded into new plates for growth curve analysis, colony formation assays or soft agar assays. Transfection of siRNAs was performed with an RNA to Lipofectamine 2000 ratio of 1:2. 72 hr post transfection cells were lysed in lysis buffer for western blot analysis [28], processed for RNA extraction or reseeded into new plates for growth curve analysis, colony formation assays or soft agar assays. Transfection of miR-18a antagomir was performed with an RNA to Lipofectamine 2000 ratio of 1:2. 24 hr post transfection cells were lysed in lysis buffer for western blot analysis [28], processed for RNA extraction or reseeded into new plates for growth curve analysis, colony formation assays or soft agar assays.

### Western blot analysis

Equal amounts of protein from cell lysates were separated by SDS PAGE and transferred onto a nitrocellulose membrane by a semi-dry transfer method (Trans Blot SD Semi-Dry Transfer cell, Bio-Rad, USA). Membranes were blocked with 5% milk solution before incubation with primary antibodies at 1:1000 dilution unless otherwise stated: p-YAP1 Ser-127 (CST; D9W2I), YAP1 (CST; D8H1X), p-MOB1 (CST; D2F1O), MOB1 (CST; E1N9D), STK4 (Abcam; ab51134) (1:2000), STK3 (Abcam; ab52641) (1:5000), Myc (9E10), Cyclin B1 (Santa Cruz Biotechnology (SCBT); sc-245), Cyclin D1 (Abcam; ab13475), Cyclin E (Thermofisher; 32–1600), HPV 16/18 E6 (CBT; sc-460), HPV 16 E7 (SCBT; sc-1587), HPV 18 E7 (Abcam; ab100953), and GAPDH (SCBT; sc365062) (1:5000) as a loading control. Horseradish peroxidase (HRP)-conjugated secondary antibodies (Sigma, USA) were used at a 1:5000 dilution. Proteins were detected using WesternBright ECL (Advansta, USA) and visualised on X-ray film.

### RNA extraction, cDNA synthesis and quantitative Real Time-PCR

Total RNA was extracted from cells using an E.Z.N.A Total RNA Kit I (omegabiotek). cDNA was synthesised using 1 μg of RNA and iScript cDNA synthesis kit (Bio Rad). qRT-PCR was performed on the synthesised cDNA on a Corbett Rotor-Gene 6000 using QuantiFast SYBR Green PCR kit (Qiagen) and analysed using the ΔΔCT method [56] normalised to the *U6* housekeeping gene. Each experiment was repeated at least 3 times. Primer sequences are in Table 1. For miScript, cDNA was synthesised using 1μg of RNA and miScript II RT Kit (Qiagen). qRT-PCR was performed using miScript SYBR Green PCR Kit (Qiagen) and analysed using the ΔΔCT method [56] normalised to SNORD68 control gene.

**Table 1:** qRT-PCR rimers used in this study.

Gene	Forward primer sequence	Reverse primer sequence
*U6*	CTCGCTTCGGCAGCACA	AACGCTTCACGCATTTGC
*STK4*	GCTTCTGACTCAATGCTTAG	CCACATCCTCCTGCCAAG
*AREG*	GTGGTGCTGTCGCTCTTGATA	ACTCACAGGGGAAATCTCACT
*Cyr61*	AGCCTCGCATCCTATACAACC	TTCTTTCACAAGGCGGCACTC
*PROM1*	TGGATGCAGAACTTGACAACGT	ATACCTGCTACGACAGTCGTGGT
*CCND1*	CCGCTGGCCATGAACTACCT	ACGAAGGTCTGCGCGTGTT
HPV16 *E6*	CTGCAATGTTTCAGGACCCAC	GTTGTTTGCAGCTCTGTGCAT
HPV16 *E7*	ATTAAATGACAGCTCAGAGGA	GCTTTGTACGCACAACCGAAGC
HPV18 *E6*	TGGCGCGCTTTGAGGA	TGTTCAGTTCCGTGCACAGATC
HPV18 *E7*	GACCTAAGGCAACATTGCA	GCTCGTGACATAGAAGGTC

### Colony formation assay

48 hr post-transfection, cells were trypsinised and reseeded in a six well plate at 500 cells per well and left to incubate for 14–21 days. Colonies were then stained (1% crystal violet, 25% methanol) and colonies were counted manually. Each experiment was repeated a minimum of 3 times.

### Soft agar assay

Cells were transfected as required. 60 mm dishes were coated with a layer of 1% agarose (ThermoFischer Scientific, USA) in 2X DMEM (ThermoFischer Scientific, USA) supplemented with 20% FBS. 48 hr post-transfection, cells were trypsinised and added to 0.7% agarose in 2X DMEM (ThermoFischer Scientific, USA) supplemented with 20% FBS at 1000 cells/mL. Once set, DMEM supplemented with 10% FBS and 50 U/mL penicillin was added. The plates were then incubated for 14–21 days. Each experiment was repeated at least three times unless stated otherwise. Visible colonies were counted manually.

### Flow cytometry

Cells were transfected as required. 48 hr post-transfection, cells were harvested and fixed in 70% ethanol overnight. The ethanol was removed and cells washed with PBS containing 0.5% (w/v) BSA. Cells were stained with PBS containing 0.5% BSA, 50 μg/mL propidium iodide (Sigma) and 5 μg/mL RNase (Sigma) and incubated in this solution for 30 min at room temperature. Samples were processed on an LSRFortessaTM cell analyzer (BD) and the PI histograms analysed on modifit software. Each experiment was repeated a minimum of 3 times.

### Microarray analysis

For microarray analysis of *STK4* expression, the following datasets were used: GSE6791, GSE9750, GSE27678, GSE39001 and GSE75132. For microarray analysis of miR-18a expression, the following datasets were used: GSE30656 and GSE86100.

### Immunofluorescence analysis

Cells were seeded onto coverslips and, 24 hr later, were transfected as required. 24 hr after transfection, cells were fixed with 4% paraformaldehyde for 10 min and then permeabilised with 0.1% (v/v) Triton for 15 minutes. Cells were then incubated in primary antibodies in PBS with 4% BSA overnight at 4ºC. Primary antibodies were used at a concentration of 1:400. Cells were washed thoroughly in PBS and then incubated with Alex-fluor conjugated secondary antibodies 594 and Alexa 488 (1:1000) (Invitrogen) in PBS with 4% BSA for 2 hours. DAPI was used to visualise nuclei. Coverslips were mounted onto slides with Prolong Gold (Invitrogen).

### Statistical analysis

Unless otherwise indicated, data was analysed using a two-tailed, unpaired Student’s t-test.

## Supporting information

S1 Fig *STK4* expression in cervical cancer cases from the GEO repository. **A)** Scatter dot plot of data acquired from the dataset GSE27678. Arbitrary values for the mRNA expression of *STK4* in normal cervix (n = 12), LSIL (n = 11) and HSIL (n = 21) samples were plotted. Normal vs LSIL, p = 0.80; Normal vs HSIL, p = 0.006; LSIL vs HSIL, p = 0.003. **B)** Scatter dot plot of data acquired from the dataset GSE75132. Arbitrary values for the mRNA expression of *STK4* in normal cervix (n = 21), LSIL (n = 4) and HSIL (n = 16) samples were plotted. Normal vs LSIL, p = 0.57; Normal vs HSIL, p = 0.007; LSIL vs HSIL, p = 0.054. **C)** Scatter dot plot of data acquired from the dataset GSE6791 on the GEO database. Arbitrary values for the mRNA expression of *STK4* in normal cervix (n = 8) and cervical cancer (n = 20) samples were plotted; Normal vs cancer, p = 0.002. **D)** Scatter dot plot of data acquired from the dataset GSE9750 on the GEO database. Arbitrary values for the mRNA expression of *STK4* in normal cervix (n = 23) and cervical cancer (n = 28) samples were plotted; Normal vs cancer, p = 0.001. **E)** Scatter dot plot of data acquired from the dataset GSE39001 on the GEO database. Arbitrary values for the mRNA expression of *STK4* in normal cervix (n = 12) and cervical cancer (n = 43) samples were plotted; Normal vs cancer, p = 0.02. Error bars represent the mean +/- standard deviation. *P<0.05, **P<0.01, ***P<0.001 (Student’s t-test).
(TIF)

S2 Fig STK4/3 inhibits proliferation and cell cycle progression in HPV16+ cervical cancer cells. **A)** Representative western blots of STK4/3 overexpression in CaSKi cells. Lysates were analysed for the phosphorylation of the STK4/3 substrate MOB1 and the downstream target YAP. The Myc epitope was used to detected successful expression of fusion proteins. GAPDH was used as a loading control. **B)** Immunofluorescence analysis of STK4/3 overexpression in CaSKi cells. Cover slips were stained for STK4/3 (red) and YAP1 (green). Nuclei were visualised using DAPI (blue). Images were acquired using identical exposure times. Scale bar, 20 μm. **C)** qPCR analysis of YAP-dependent genes (*AREG*, *PROM1*, *Cyr61* and *CNND1)* in CaSKi cells overexpressing STK4/3. *U6* expression was used as a loading control (n = 3). **D)** Growth curve analysis of CaSKi cells overexpressing STK4/3. **E)** Colony formation assay (anchorage dependent growth) of CaSKi cells overexpressing STK4/3 (n = 3). **F)** Soft agar assay (anchorage independent growth) of CaSKi cells overexpressing STK4/3 (n = 3). **G)** Representative western blots of CaSKi cells overexpressing STK4/3 analysed for the expression of cyclin proteins. The Myc epitope was used to detect successful expression of fusion proteins. GAPDH was used as a loading control. **H)** Flow cytometric analysis of cell cycle profile of CaSKi cells overexpressing STK4/3. Error bars represent the mean +/- standard deviation of a minimum of three biological repeats. *P<0.05, **P<0.01, ***P<0.001 (Student’s t-test).
(TIF)

S3 Fig STK4/3 does not inhibit proliferation and cell cycle progression in C33A cells. **A)** Representative western blots of STK4/3 overexpression in C33A cells. Lysates were analysed for the phosphorylation of the STK4/3 substrate MOB1 and the downstream target YAP. The Myc epitope was used to detect successful expression of fusion proteins. GAPDH was used as a loading control. **B)** Immunofluorescence analysis of STK4/3 overexpression in C33A cells. Cover slips were stained for STK4/3 (red) and YAP (green). Nuclei were visualised using DAPI (blue). Images were acquired using identical exposure times. Scale bar, 20 μm. **C)** qPCR analysis of YAP-dependent genes (*AREG*, *PROM1*, *Cyr61* and *CNND1)* in C33A cells overexpressing STK4/3. *U6* expression was used as a loading control (n = 3). **D)** Growth curve analysis of C33A cells overexpressing STK4/3. **E)** Colony formation assay (anchorage dependent growth) of C33A cells overexpressing STK4/3 (n = 3). **F)** Flow cytometric analysis of cell cycle profile of C33A cells overexpressing STK4/3. Error bars represent the mean +/- standard deviation of a minimum of three biological repeats. *P<0.05, **P<0.01, ***P<0.001 (Student’s t-test).
(TIF)

S4 Fig Inhibition of STK4/3 kinase activity prevents the block on proliferation and tumourigenesis in HPV16+ cervical cancer cells. **A)** Representative western blots of STK4/3 overexpression in CaSKi cells with or without treatment with XMU-MP1 for 8 hours prior to lysis. Lysates were analysed for the phosphorylation of the STK4/3 substrate MOB1, the downstream target YAP and the YAP target gene cyclin D1. GAPDH was used as a loading control. **B)** Immunofluorescence analysis of STK4/3 overexpression in CaSKi cells with or without treatment with XMU-MP1 for 8 hours prior to analysis. Cover slips were stained for STK4/3 (red) and YAP1 (green). Nuclei were visualised using DAPI (blue). Images were acquired using identical exposure times. Scale bar, 20 μm. **C)** Growth curve analysis of CaSKi cells overexpressing STK4/3 with or without treatment with XMU-MP1 for 8 hours prior to re-seeding (n = 3). **D)** Colony formation assay (anchorage dependent growth) of CaSKi cells overexpressing STK4/3 with or without treatment with XMU-MP1 for 8 hours prior to re-seeding (n = 3). **E)** Soft agar assay (anchorage independent growth) of CaSKi cells overexpressing STK4/3 with or without treatment with XMU-MP1 for 8 hours prior to re-seeding. Error bars represent the mean +/- standard deviation of a minimum of three biological repeats. *P<0.05, **P<0.01, ***P<0.001 (Student’s t-test).
(TIF)

S5 Fig Kinase null STK4/3 mutants cannot inhibit proliferation and tumourigenesis in HPV16+ cervical cancer cells. **A)** Representative western blots of STK4/3 or KD STK4/3 overexpression in CaSKi cells. Lysates were analysed for the phosphorylation of the STK4/3 substrate MOB1, the downstream target YAP and the YAP target gene cyclin D1. Antibodies against STK4/3 were used to detect successful expression of fusion proteins. GAPDH was used as a loading control. **B)** Immunofluorescence analysis of STK4/3 or KD STK4/3 overexpression in CaSKi cells. Cover slips were stained for STK4/3 (red) and YAP (green). Nuclei were visualised using DAPI (blue). Images were acquired using identical exposure times. Scale bar, 20 μm. **C)** Growth curve analysis of CaSKi cells overexpressing STK4/3 or KD STK4/3 (n = 3). **D)** Colony formation assay (anchorage dependent growth) of CaSKi cells overexpressing STK4/3 or KD STK4/3 (n = 3). **E)** Soft agar assay (anchorage independent growth) of CaSKi cells overexpressing STK4/3 or KD STK4/3 (n = 3). Error bars represent the mean +/- standard deviation of a minimum of three biological repeats. *P<0.05, **P<0.01, ***P<0.001 (Student’s t-test).
(TIF)

S6 Fig Genotoxic stress does not induce *STK4* expression. **A)** qPCR analysis of *STK4* expression in HeLa cells treated with Staurosporine (50 μM) for 48 hours. *U6* expression was used as a loading control (n = 3). **B)** qPCR analysis of *STK4* expression in HeLa cells treated with H_2_O_2_ (200 μM) for 48 hours. *U6* expression was used as a loading control (n = 3). **C)** qPCR analysis of *STK4* expression in HeLa cells treated with Etoposide (2 μM) for 48 hours. *U6* expression was used as a loading control (n = 3). **D)** qPCR analysis of *STK4* expression in HeLa cells transfected with pcDNA-p53 for 48 hours. *U6* expression was used as a loading control (n = 3). **E)** Representative western blot of HeLa cells transfected with pcDNA-p53 for 48 hours. Cell lysates were probed for the expression of p53. GAPDH was used as a loading control. Error bars represent the mean +/- standard deviation of a minimum of three biological repeats. *P<0.05, **P<0.01, ***P<0.001.
(TIF)

S7 Fig miR-18a directly regulates *STK4* in HPV16+ cervical cancer cells. **A)** Luciferase reporter assays from CaSKi cells cotransfected with miR-18a antagomir and either a wild-type *STK4* 3’UTR reporter plasmid or a mutant that lacks the putative miR-18a binding site. Data presented are relative to an internal firefly luciferase control (n = 3). **B)** miScript analysis of miR-18a levels in CaSKi transfected with specific E6/E7 siRNA with or without a miR-18a mimic. snORD68 was used as a loading control. *STK4* expression was also analysed. *U6* was used as a loading control (n = 3). **C)** Luciferase reporter assays from CaSKi cells cotransfected with specific E6/E7 siRNA, with or without a miR-18a mimic, and either a wild-type *STK4* 3’UTR reporter plasmid or a mutant that lacks the putative miR-18a binding site. Data presented are relative to an internal firefly luciferase control (n = 3). **D)** Representative western blot of CaSKi cells cotransfected with specific E6/E7 siRNA, with or without a miR-18a mimic. Cell lysates were probed for the expression of STK4. GAPDH was used as a loading control. Error bars represent the mean +/- standard deviation of a minimum of three biological repeats. *P<0.05, **P<0.01, ***P<0.001.
(TIF)

S8 Fig miR-18a does not effect *STK4* in C33A cells. **A)** qPCR analysis of *STK4* transcript levels in C33A cells transfected with an miR-18a antagomir (80 nM). *U6* was used as a loading control (n = 3). Error bars represent the mean +/- standard deviation of a minimum of three biological repeats. *P<0.05, **P<0.01, ***P<0.001.
(TIF)
