# Supplementary figures and images for: MicroRNA-18a targeting of the STK4/MST1 tumour suppressor is necessary for transformation in HPV positive cervical cancer
Source: PLoS Pathog. 2020 Jun 18;16(6):e1008624. doi: 10.1371/journal.ppat.1008624 (PMC7326282; doi:10.1371/journal.ppat.1008624)

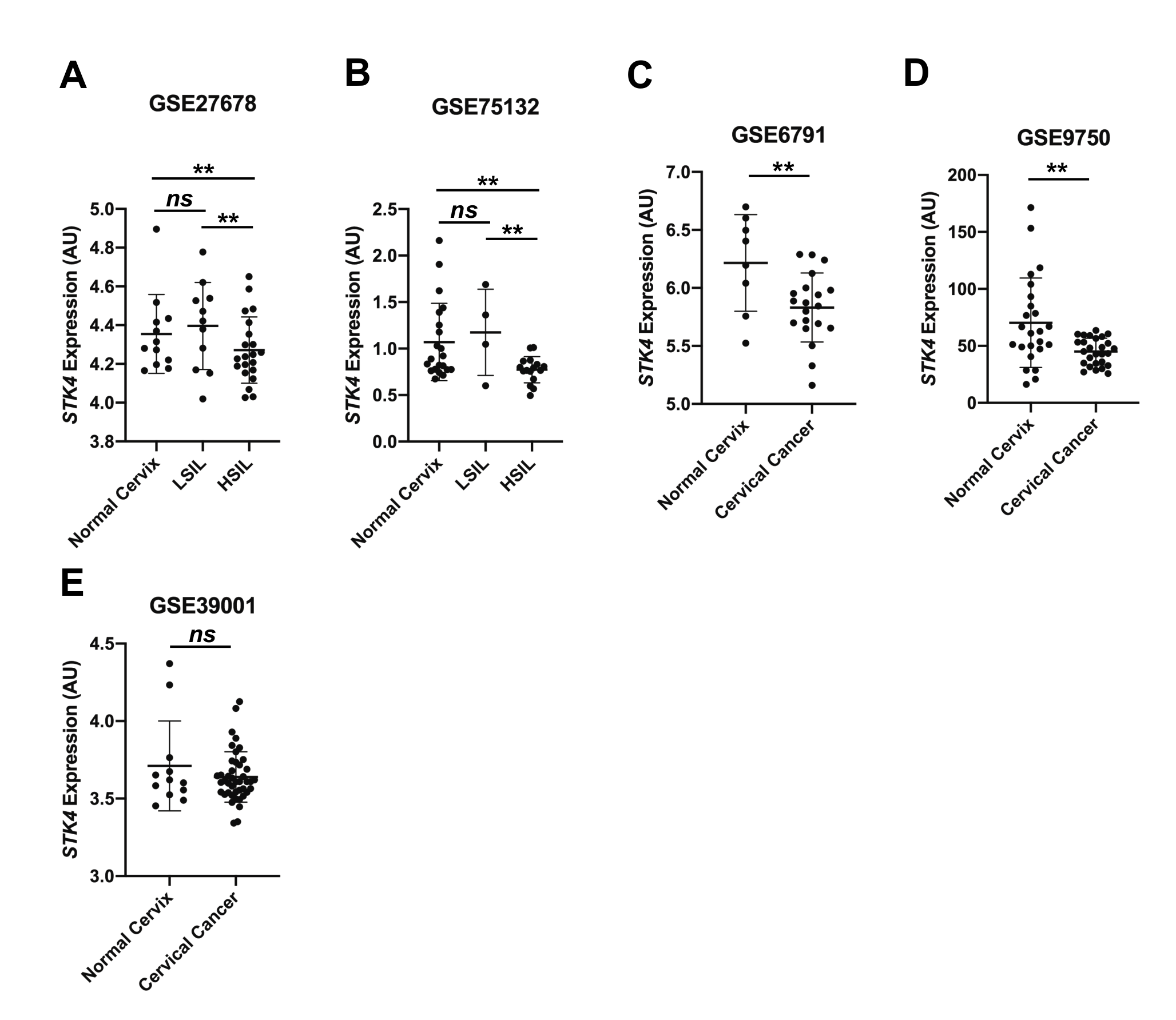

Supplement: S1 Fig — A) Scatter dot plot of data acquired from the dataset GSE27678. Arbitrary values for the mRNA expression of STK4 in normal cervix (n = 12), LSIL (n = 11) and HSIL (n = 21) samples were plotted. Normal vs LSIL, p = 0.80; Normal vs HSIL, p = 0.006; LSIL vs HSIL, p = 0.003. B) Scatter dot plot of data acquired from the dataset GSE75132. Arbitrary values for the mRNA expression of STK4 in normal cervix (n = 21), LSIL (n = 4) and HSIL (n = 16) samples were plotted. Normal vs LSIL, p = 0.57; Normal vs HSIL, p = 0.007; LSIL vs HSIL, p = 0.054. C) Scatter dot plot of data acquired from the dataset GSE6791 on the GEO database. Arbitrary values for the mRNA expression of STK4 in normal cervix (n = 8) and cervical cancer (n = 20) samples were plotted; Normal vs cancer, p = 0.002. D) Scatter dot plot of data acquired from the dataset GSE9750 on the GEO database. Arbitrary values for the mRNA expression of STK4 in normal cervix (n = 23) and cervical cancer (n = 28) samples were plotted; Normal vs cancer, p = 0.001. E) Scatter dot plot of data acquired from the dataset GSE39001 on the GEO database. Arbitrary values for the mRNA expression of STK4 in normal cervix (n = 12) and cervical cancer (n = 43) samples were plotted; Normal vs cancer, p = 0.02. Error bars represent the mean +/- standard deviation. *P<0.05, **P<0.01, ***P<0.001 (Student’s t-test). (TIF) [file ppat.1008624.s001.tif]

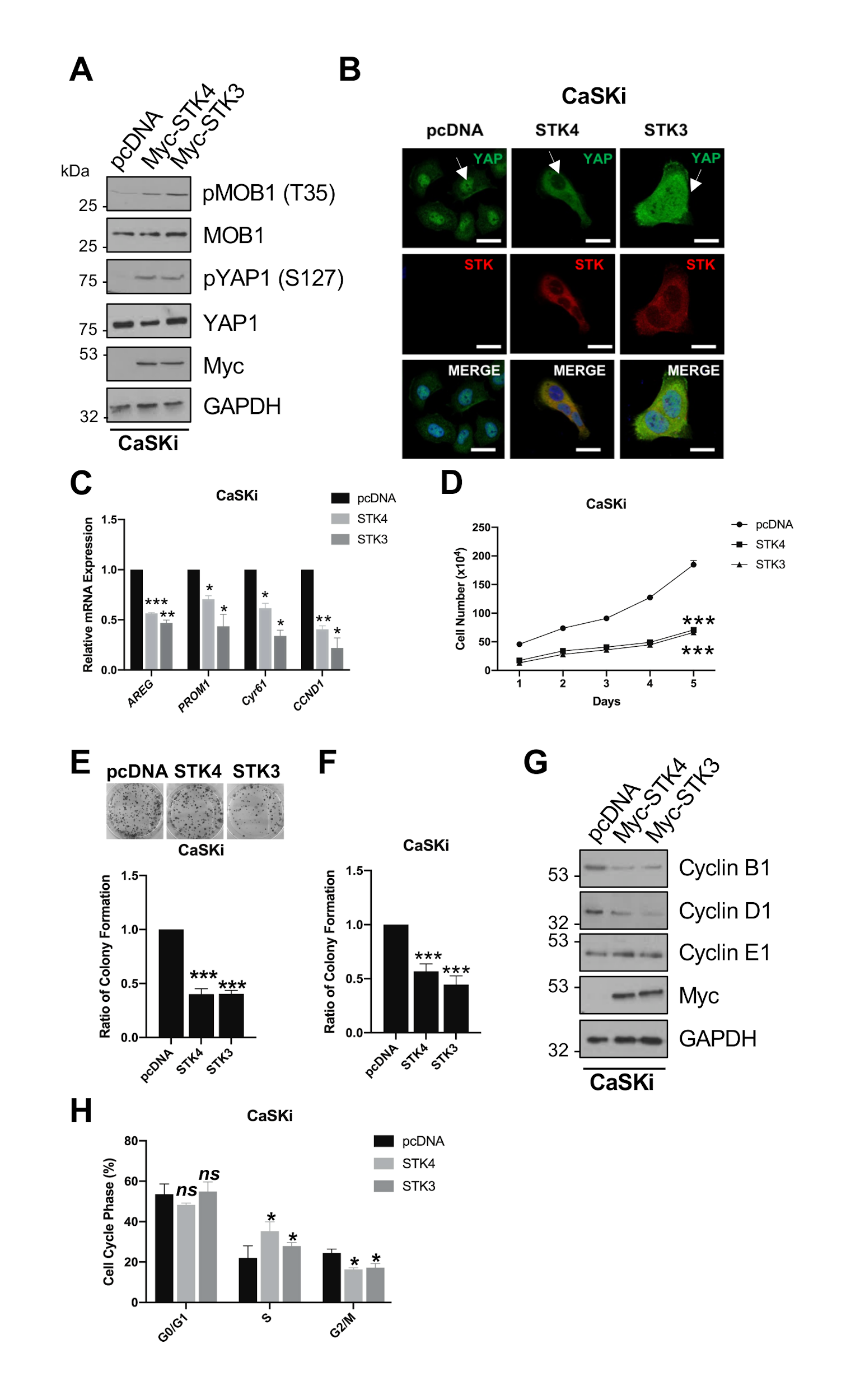

Supplement: S2 Fig — A) Representative western blots of STK4/3 overexpression in CaSKi cells. Lysates were analysed for the phosphorylation of the STK4/3 substrate MOB1 and the downstream target YAP. The Myc epitope was used to detected successful expression of fusion proteins. GAPDH was used as a loading control. B) Immunofluorescence analysis of STK4/3 overexpression in CaSKi cells. Cover slips were stained for STK4/3 (red) and YAP1 (green). Nuclei were visualised using DAPI (blue). Images were acquired using identical exposure times. Scale bar, 20 μm. C) qPCR analysis of YAP-dependent genes (AREG, PROM1, Cyr61 and CNND1) in CaSKi cells overexpressing STK4/3. U6 expression was used as a loading control (n = 3). D) Growth curve analysis of CaSKi cells overexpressing STK4/3. E) Colony formation assay (anchorage dependent growth) of CaSKi cells overexpressing STK4/3 (n = 3). F) Soft agar assay (anchorage independent growth) of CaSKi cells overexpressing STK4/3 (n = 3). G) Representative western blots of CaSKi cells overexpressing STK4/3 analysed for the expression of cyclin proteins. The Myc epitope was used to detect successful expression of fusion proteins. GAPDH was used as a loading control. H) Flow cytometric analysis of cell cycle profile of CaSKi cells overexpressing STK4/3. Error bars represent the mean +/- standard deviation of a minimum of three biological repeats. *P<0.05, **P<0.01, ***P<0.001 (Student’s t-test). (TIF) [file ppat.1008624.s002.tif]

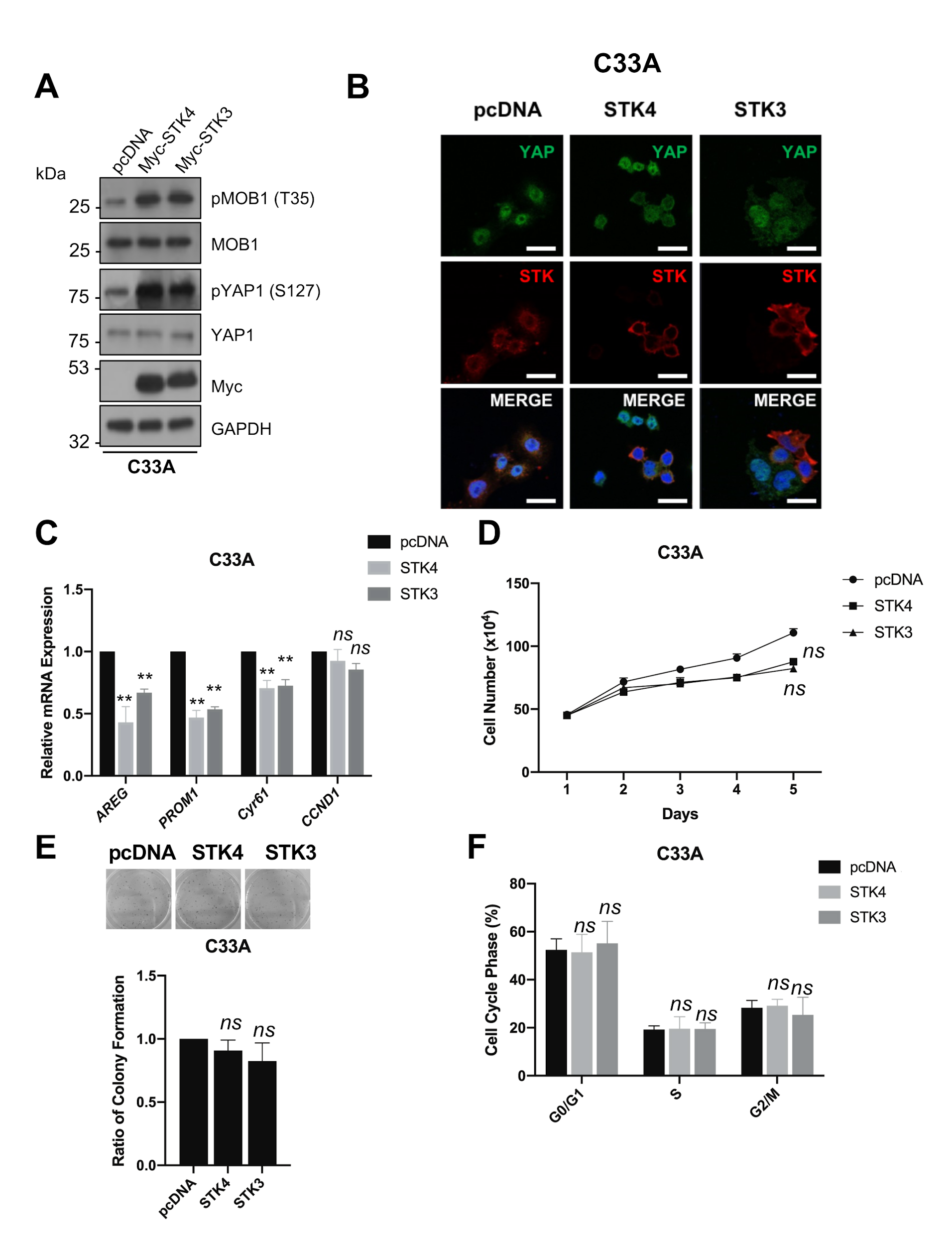

Supplement: S3 Fig — A) Representative western blots of STK4/3 overexpression in C33A cells. Lysates were analysed for the phosphorylation of the STK4/3 substrate MOB1 and the downstream target YAP. The Myc epitope was used to detect successful expression of fusion proteins. GAPDH was used as a loading control. B) Immunofluorescence analysis of STK4/3 overexpression in C33A cells. Cover slips were stained for STK4/3 (red) and YAP (green). Nuclei were visualised using DAPI (blue). Images were acquired using identical exposure times. Scale bar, 20 μm. C) qPCR analysis of YAP-dependent genes (AREG, PROM1, Cyr61 and CNND1) in C33A cells overexpressing STK4/3. U6 expression was used as a loading control (n = 3). D) Growth curve analysis of C33A cells overexpressing STK4/3. E) Colony formation assay (anchorage dependent growth) of C33A cells overexpressing STK4/3 (n = 3). F) Flow cytometric analysis of cell cycle profile of C33A cells overexpressing STK4/3. Error bars represent the mean +/- standard deviation of a minimum of three biological repeats. *P<0.05, **P<0.01, ***P<0.001 (Student’s t-test). (TIF) [file ppat.1008624.s003.tif]

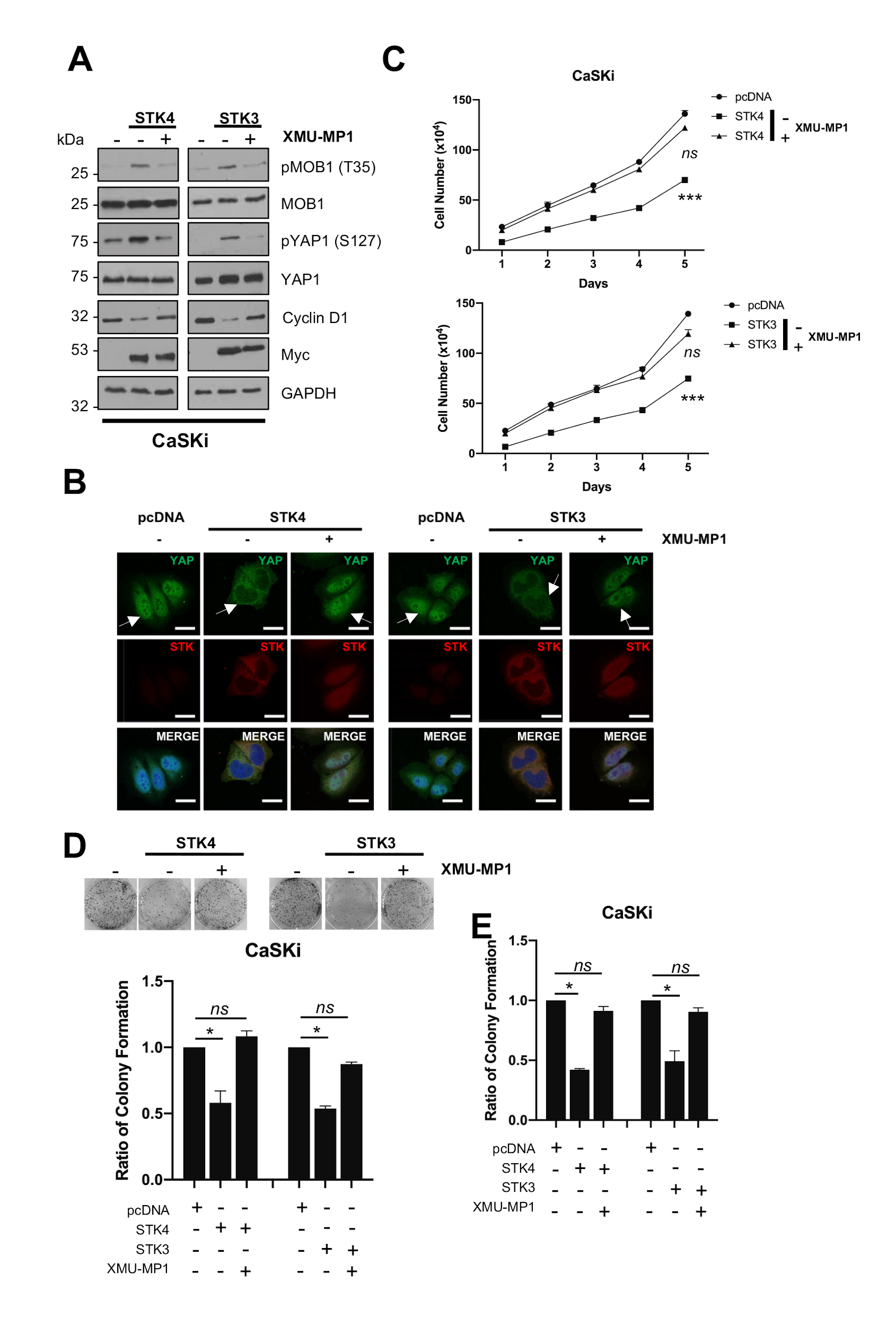

Supplement: S4 Fig — A) Representative western blots of STK4/3 overexpression in CaSKi cells with or without treatment with XMU-MP1 for 8 hours prior to lysis. Lysates were analysed for the phosphorylation of the STK4/3 substrate MOB1, the downstream target YAP and the YAP target gene cyclin D1. GAPDH was used as a loading control. B) Immunofluorescence analysis of STK4/3 overexpression in CaSKi cells with or without treatment with XMU-MP1 for 8 hours prior to analysis. Cover slips were stained for STK4/3 (red) and YAP1 (green). Nuclei were visualised using DAPI (blue). Images were acquired using identical exposure times. Scale bar, 20 μm. C) Growth curve analysis of CaSKi cells overexpressing STK4/3 with or without treatment with XMU-MP1 for 8 hours prior to re-seeding (n = 3). D) Colony formation assay (anchorage dependent growth) of CaSKi cells overexpressing STK4/3 with or without treatment with XMU-MP1 for 8 hours prior to re-seeding (n = 3). E) Soft agar assay (anchorage independent growth) of CaSKi cells overexpressing STK4/3 with or without treatment with XMU-MP1 for 8 hours prior to re-seeding. Error bars represent the mean +/- standard deviation of a minimum of three biological repeats. *P<0.05, **P<0.01, ***P<0.001 (Student’s t-test). (TIF) [file ppat.1008624.s004.tif]

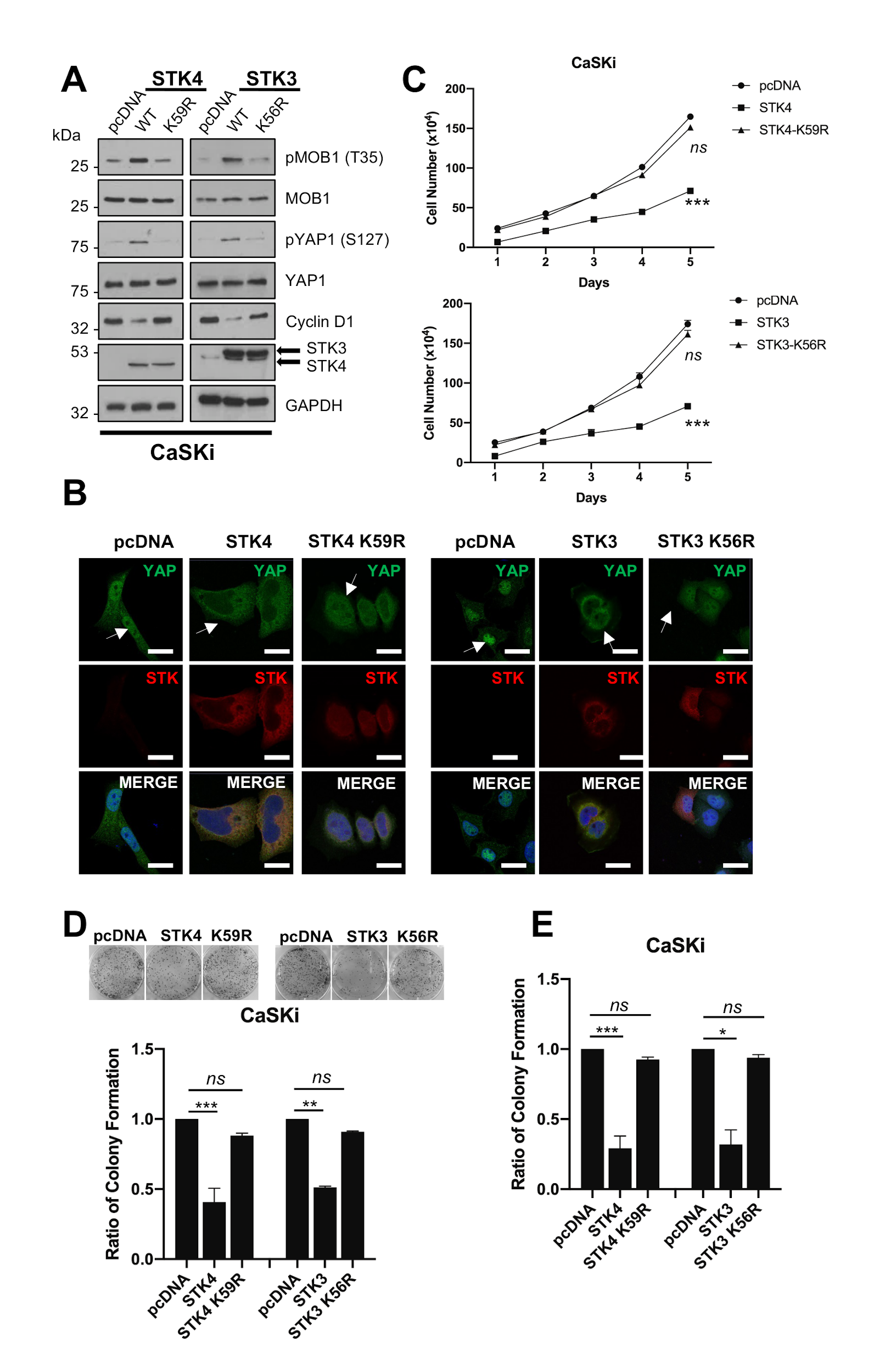

Supplement: S5 Fig — A) Representative western blots of STK4/3 or KD STK4/3 overexpression in CaSKi cells. Lysates were analysed for the phosphorylation of the STK4/3 substrate MOB1, the downstream target YAP and the YAP target gene cyclin D1. Antibodies against STK4/3 were used to detect successful expression of fusion proteins. GAPDH was used as a loading control. B) Immunofluorescence analysis of STK4/3 or KD STK4/3 overexpression in CaSKi cells. Cover slips were stained for STK4/3 (red) and YAP (green). Nuclei were visualised using DAPI (blue). Images were acquired using identical exposure times. Scale bar, 20 μm. C) Growth curve analysis of CaSKi cells overexpressing STK4/3 or KD STK4/3 (n = 3). D) Colony formation assay (anchorage dependent growth) of CaSKi cells overexpressing STK4/3 or KD STK4/3 (n = 3). E) Soft agar assay (anchorage independent growth) of CaSKi cells overexpressing STK4/3 or KD STK4/3 (n = 3). Error bars represent the mean +/- standard deviation of a minimum of three biological repeats. *P<0.05, **P<0.01, ***P<0.001 (Student’s t-test). (TIF) [file ppat.1008624.s005.tif]

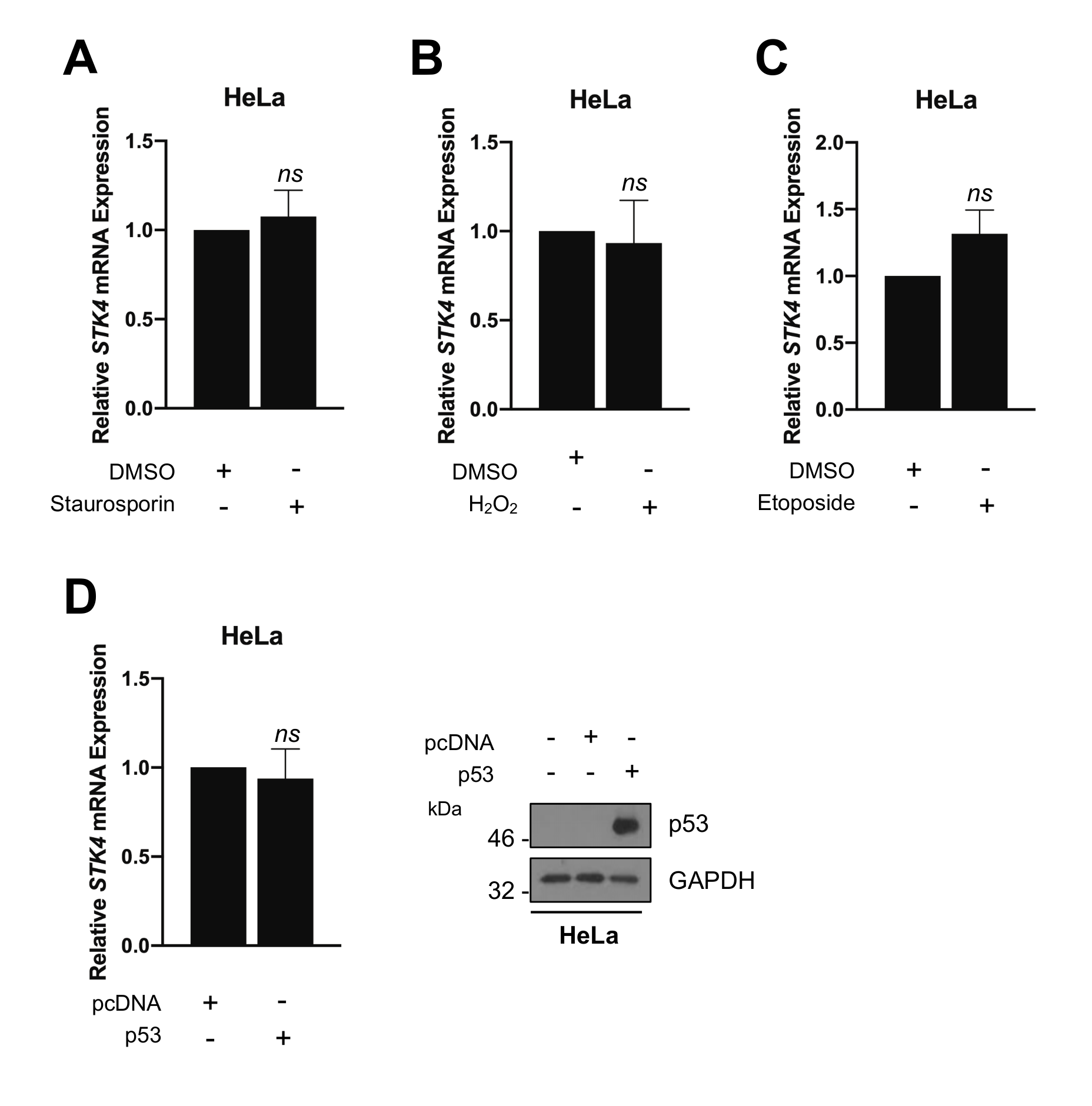

Supplement: S6 Fig — A) qPCR analysis of STK4 expression in HeLa cells treated with Staurosporine (50 μM) for 48 hours. U6 expression was used as a loading control (n = 3). B) qPCR analysis of STK4 expression in HeLa cells treated with H2O2 (200 μM) for 48 hours. U6 expression was used as a loading control (n = 3). C) qPCR analysis of STK4 expression in HeLa cells treated with Etoposide (2 μM) for 48 hours. U6 expression was used as a loading control (n = 3). D) qPCR analysis of STK4 expression in HeLa cells transfected with pcDNA-p53 for 48 hours. U6 expression was used as a loading control (n = 3). E) Representative western blot of HeLa cells transfected with pcDNA-p53 for 48 hours. Cell lysates were probed for the expression of p53. GAPDH was used as a loading control. Error bars represent the mean +/- standard deviation of a minimum of three biological repeats. *P<0.05, **P<0.01, ***P<0.001. (TIF) [file ppat.1008624.s006.tif]

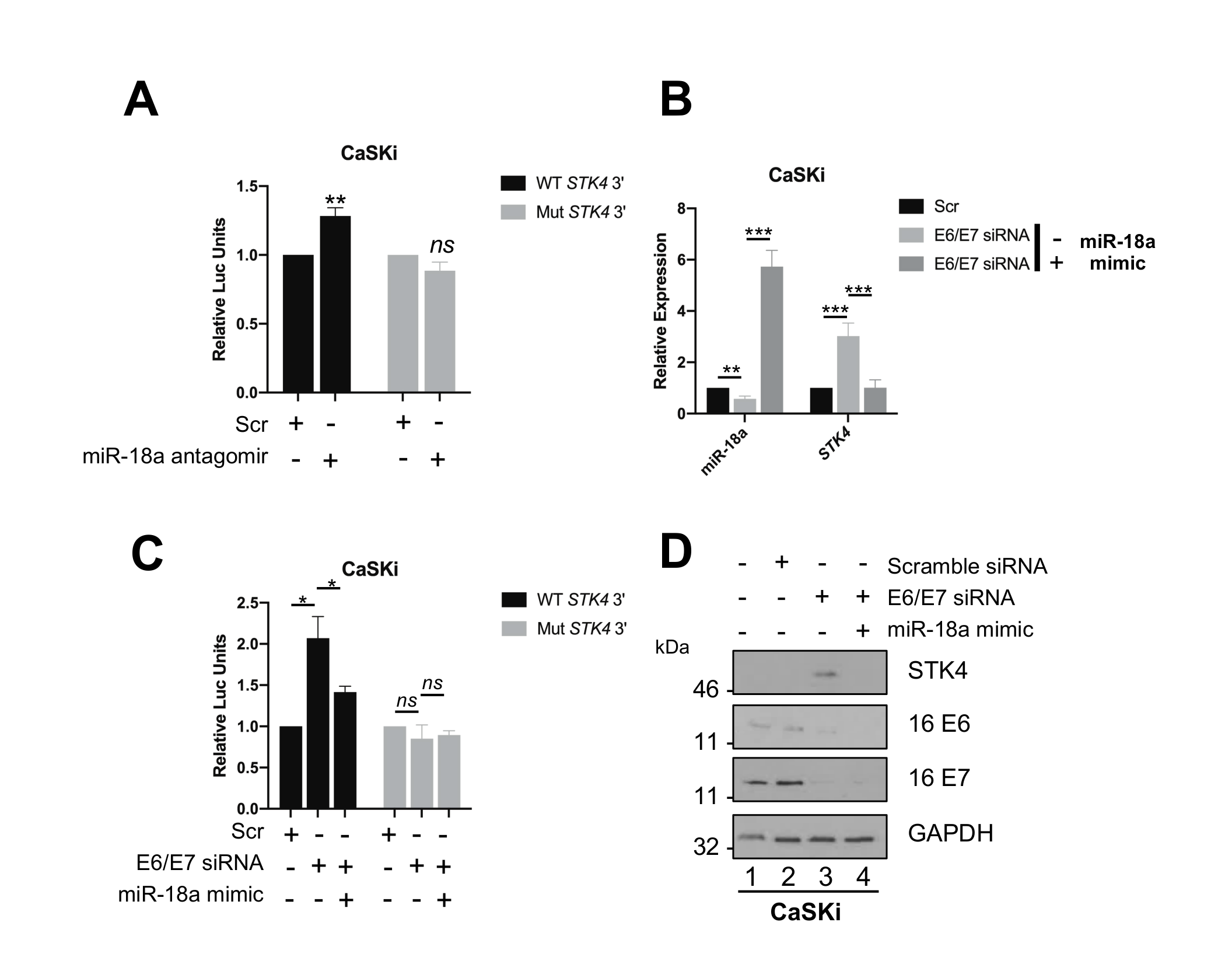

Supplement: S7 Fig — A) Luciferase reporter assays from CaSKi cells cotransfected with miR-18a antagomir and either a wild-type STK4 3’UTR reporter plasmid or a mutant that lacks the putative miR-18a binding site. Data presented are relative to an internal firefly luciferase control (n = 3). B) miScript analysis of miR-18a levels in CaSKi transfected with specific E6/E7 siRNA with or without a miR-18a mimic. snORD68 was used as a loading control. STK4 expression was also analysed. U6 was used as a loading control (n = 3). C) Luciferase reporter assays from CaSKi cells cotransfected with specific E6/E7 siRNA, with or without a miR-18a mimic, and either a wild-type STK4 3’UTR reporter plasmid or a mutant that lacks the putative miR-18a binding site. Data presented are relative to an internal firefly luciferase control (n = 3). D) Representative western blot of CaSKi cells cotransfected with specific E6/E7 siRNA, with or without a miR-18a mimic. Cell lysates were probed for the expression of STK4. GAPDH was used as a loading control. Error bars represent the mean +/- standard deviation of a minimum of three biological repeats. *P<0.05, **P<0.01, ***P<0.001. (TIF) [file ppat.1008624.s007.tif]

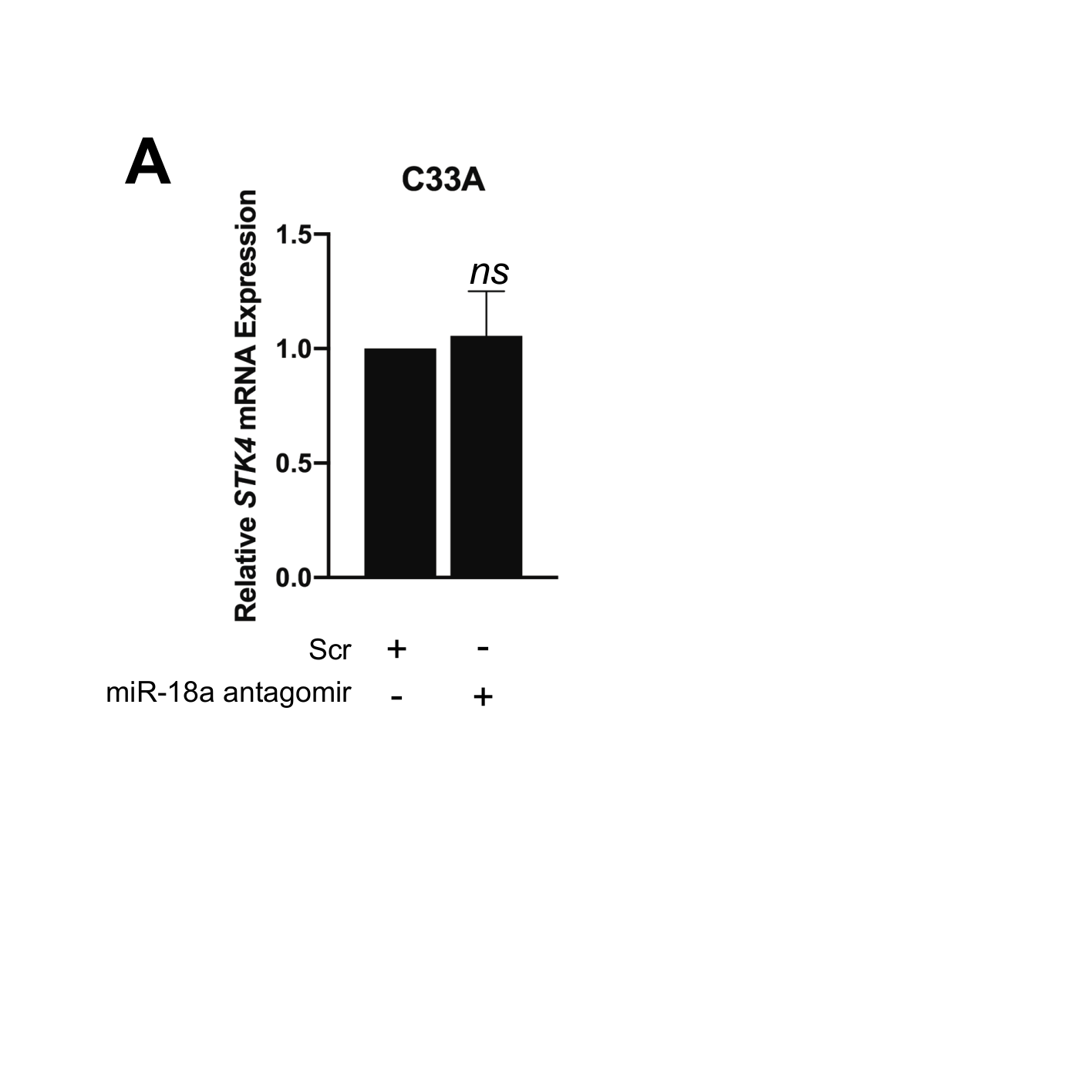

Supplement: S8 Fig — A) qPCR analysis of STK4 transcript levels in C33A cells transfected with an miR-18a antagomir (80 nM). U6 was used as a loading control (n = 3). Error bars represent the mean +/- standard deviation of a minimum of three biological repeats. *P<0.05, **P<0.01, ***P<0.001. (TIF) [file ppat.1008624.s008.tif]
